# Characterization of an AA9 LPMO from *Thielavia australiensis*, *Taus*LPMO9B, under industrially relevant lignocellulose saccharification conditions

**DOI:** 10.1186/s13068-020-01836-3

**Published:** 2020-11-30

**Authors:** F. Calderaro, M. Keser, M. Akeroyd, L. E. Bevers, V. G. H. Eijsink, A. Várnai, M. A. van den Berg

**Affiliations:** 1 DSM Biotechnology Center, PP 699-0310, Alexander Fleminglaan 1, 2613 AX Delft, The Netherlands; 2 Molecular Enzymology, Groningen Biomolecular Sciences and Biotechnology Institute, University of Groningen, Groningen, The Netherlands; 3 Faculty of Chemistry, Biotechnology and Food Science, Norwegian University of Life Sciences (NMBU), Ås, Norway

**Keywords:** Lytic polysaccharide monooxygenase, Enzyme assay, Cellulose, Lignocellulosic biomass, *Thielavia australiensis*, Corn stover, H_2_O_2_

## Abstract

**Background:**

The discovery of lytic polysaccharide monooxygenases (LPMO) has changed our perspective on enzymatic degradation of plant biomass. Through an oxidative mechanism, these enzymes are able to cleave and depolymerize various polysaccharides, acting not only on crystalline substrates such as chitin and cellulose, but also on other polysaccharides, such as xyloglucan, glucomannan and starch. Despite their widespread use, uncertainties related to substrate specificity and stereospecificity, the nature of the co-substrate, in-process stability, and the nature of the optimal reductant challenge their exploitation in biomass processing applications.

**Results:**

In this work, we studied the properties of a novel fungal LPMO from the thermophilic fungus *Thielavia australiensis, Taus*LPMO9B. Heterologous expression of *Taus*LPMO9B in *Aspergillus niger* yielded a glycosylated protein with a methylated N-terminal histidine showing LPMO activity. High sequence identity of the AA9 domain to that of *Mt*LPMO9B (MYCTH_80312) from *Myceliophthora thermophila* (84%) indicated strictly C1-oxidizing activity on cellulose, which was confirmed experimentally by the analysis of products released from cellulose using HPAEC. The enzyme was stable and active at a pH ranging from 4 to 6, thus matching the conditions commonly used in industrial biomass processing, where a low pH (between 4 and 5) is used due to the pH-optima of commercial cellulases and a desire to limit microbial contamination.

**Conclusion:**

While the oxidative cleavage of phosphoric acid swollen cellulose (PASC) by *Taus*LPMO9B was boosted by the addition of H_2_O_2_ as a co-substrate, this effect was not observed during the saccharification of acid pretreated corn stover. This illustrates key differences between the lab-scale tests with artificial, lignin-free substrates and industrial settings with lignocellulosic biomass as substrate.

## Background

Due to the increasing demand for renewable alternatives to fossil resources for the production of fuels and chemicals, the attention towards the utilization of lignocellulosic material to generate second-generation biofuels has grown exponentially in recent years [1, 2]. Despite the abundance of lignocellulosic material, constituting the structural part of the plant cell wall, its utilization as an industrial feedstock poses some challenges, largely due to its recalcitrant nature [3, 4]. Lignocellulose is mainly composed of cellulose, hemicellulose and lignin, closely interlinked together to form a strong matrix that, in nature, provides plants with resistance to mechanical and microbial attack [5]. Although the polysaccharides constituting (hemi)cellulose can be enzymatically converted into fermentable sugars to produce biofuels, the tightly packed structure of the plant cell wall hinders the accessibility of enzymes to the substrate. Therefore, chemical and/or physical pretreatments are necessary to allow opening of this structure [6]. Next, an efficient enzymatic cocktail to break down the plant cell wall structure and release monosaccharides, is key in making the production of second-generation biofuels a commercially viable process [7]. Hydrolysis of cellulose, in particular, relies on the synergistic action of a mixture of different enzymes. Cellobiohydrolases and endoglucanases cleave, respectively, at the end or in the internal part of the cellulose chains and release cellobiose and longer cello-oligosaccharides, which are finally hydrolyzed to glucose by β-glucosidases [4]. These enzymes are classified as glycoside hydrolases (GH) in the CAZy (Carbohydrate Active Enzyme) database [8], based on sequence similarity.

Lytic polysaccharide monooxygenases (LPMOs) have been discovered to contribute to the deconstruction of recalcitrant polysaccharides by conventional GHs in these enzyme cocktails. With their oxidative nature, LPMOs have changed the classical view on cellulose depolymerization as a process solely depending on hydrolytic enzymes [9]. Due to their ability to boost the efficiency of cellulolytic enzymes in the saccharification of lignocellulosic biomass [10, 11], LPMOs form today a crucial component in industrial enzymatic preparations [12]. To optimize the saccharification step, it is necessary to assess the biochemical properties of the involved enzymes under relevant process conditions and select the most efficient enzymes and process conditions for a particular process. While the traditional GHs have already been known for decades and have been extensively studied, LPMOs are a class of enzymes whose features, including their kinetics and stability during lignocellulose hydrolysis, still need to be fully elucidated to optimize their utilization in an industrial setting [13, 14].

Fungal cellulose-active LPMOs were formerly classified as GH61 endoglucanase. After the discovery of their true nature [9], GH61s were reclassified as family 9 auxiliary activities (AAs) in the CAZy database. There are currently seven AA families in the CAZy database (AA9–AA11, AA13–AA16) that comprise LPMOs from different sources, including bacteria, plant, fungi and insects [15]. The LPMO reaction starts with the reduction of the active-site copper from Cu^2+^ to Cu^+^ by an external electron donor, which can be a small molecule, such as ascorbic acid, or an enzyme, such as cellobiose dehydrogenase [16–18]. Of note, electron donors can drive LPMO reaction to varying extents [16, 19]; thus, the selection of electron donor will determine apparent enzyme efficiency. Several studies have shown that reduction of LPMOs acting on lignin-rich lignocellulosic material does not require addition of external electron donors [19–24], suggesting a key impact of lignin on LPMO catalytic performance. The reduction step is followed by a reaction with an oxygen co-substrate, originally proposed to be O_2_ [9], that will eventually result in the hydroxylation of either the C1 or C4 carbon in the glycosidic bond of the polysaccharide. The hydroxylated glycosidic bond is unstable and will break [25], leading to the formation of an aldonolactone (C1-oxidation) or a 4-ketoaldose (C4-oxidation) [26, 27]. In 2017, the O_2_-driven mechanism was challenged, by proposing H_2_O_2_ as the relevant co-substrate for LPMO [18]. Since then, there is growing evidence in the literature in support of the H_2_O_2_-driven mechanism [23, 28–31]. While in both cases, activation of the enzyme depends on enzyme reduction, the O_2_-driven reaction requires delivery of two electrons per catalytic cycle, whereas a reduced LPMO can catalyze multiple reactions with H_2_O_2_ without any further electron supply.

LPMOs are quite abundant in filamentous fungi [32], but the biological role of this abundance remains unclear. There are indications that LPMOs may have evolved to adapt to various plant cell wall substructures and electron donors [33]. Intriguingly, the number of LPMOs in (thermophilic) filamentous fungi and their expression levels vary to a great extent. As an example, *Thielavia australiensis* encodes 15 AA9 LPMOs in its genome (Genozyme (http://genome.fungalgenomics.ca/; [34]).

Industrial enzyme cocktails are generally produced in filamentous fungal systems [35, 36]. On the other hand, many of the fungal LPMOs studied to date have been produced in *Pichia pastoris*, a preferred expression system for detailed enzyme characterization because of a low background of plant cell wall-active CAZymes. Notably, LPMOs expressed in *P. pastoris* carry different post-translational modifications (PTMs), including the lack of methylation at the N-terminal His [37] and different glycosylation patterns, compared to those expressed in fungal hosts. While the individual effects of these PTMs on LPMO activity are little understood, Petrović et al. have shown recently that the (methylated) variant of *Ta*LPMO9A produced in *Aspergillus niger* has higher functional stability than a (non-methylated) variant produced in *P. pastoris* [38]. Concerning glycosylations, some studies have suggested that N-glycosylation near the catalytic site could be involved, in some cases, in substrate binding of LPMOs [39]. Glycosylation, in certain cases, may complement substrate binding by cellulose-specific Carbohydrate-Binding Modules (CBMs). Despite the role of these modifications still being uncertain, producing LPMOs in a filamentous fungal host ensures that the produced enzymes are representative for the ones that will be used in commercial applications.

For industrial biomass processing, conducting industrial enzymatic saccharification at higher temperature brings many advantages, such as reduced risk of microbial contaminations or reduced viscosity of the material. Thermophilic fungi, defined as fungi able to grow at temperatures of 50 °C or above [40, 41], represent an interesting source of potentially thermostable enzymes. So far, different LPMOs from thermophilic/thermotolerant fungi have been characterized, including enzymes from *Myceliophthora thermophila* [19], *Thermoascus aurantiacus* [38], *Thielavia terrestris* [10] and *Malbranchea cinnamomea* [42]. Another aspect in the discovery of novel LPMOs is their ability to perform well under conditions that are compatible with the commonly used acidic pretreatment (pH between 2 and 3) of lignocellulosic material. Thus, LPMOs from acid-tolerant, thermophilic filamentous fungi are of interest. This study focused on the characterization of such an LPMO, *Taus*LPMO9B, from *T. australiensis*, which is, together with *T. terrestris,* the only described thermophilic species of the genus *Thielavia* [41]. *Taus*LPMO9B was identified as part of a fungal secretome analysis project. *Taus*LPMO9B was expressed in *A. niger*, an industrially relevant production organism, and assessed in terms of substrate specificity, oxidative regiospecificity, temperature and pH dependency, electron donor specificity, co-substrate utilization (H_2_O_2_ and O_2_) and performance during biomass degradation. As lignocellulosic feedstock is a complex material, it is rather challenging to use it as a substrate for biochemical characterization of an LPMO: in the presence of a cellulase mix, C1-oxidized products are converted to gluconic acid and cellobionic acid, which are difficult to discriminate from other products with a similar elution profiles. Therefore, we also included phosphoric acid swollen cellulose (PASC) as a cleaner, non-natural substrate to allow for more detailed biochemical studies, and we assessed activity on this model cellulose (PASC) as well as hemicelluloses (isolated hemicelluloses), in addition to pretreated corn stover.

## Results

### Sequence analysis and homology model

**Fig. 1:**
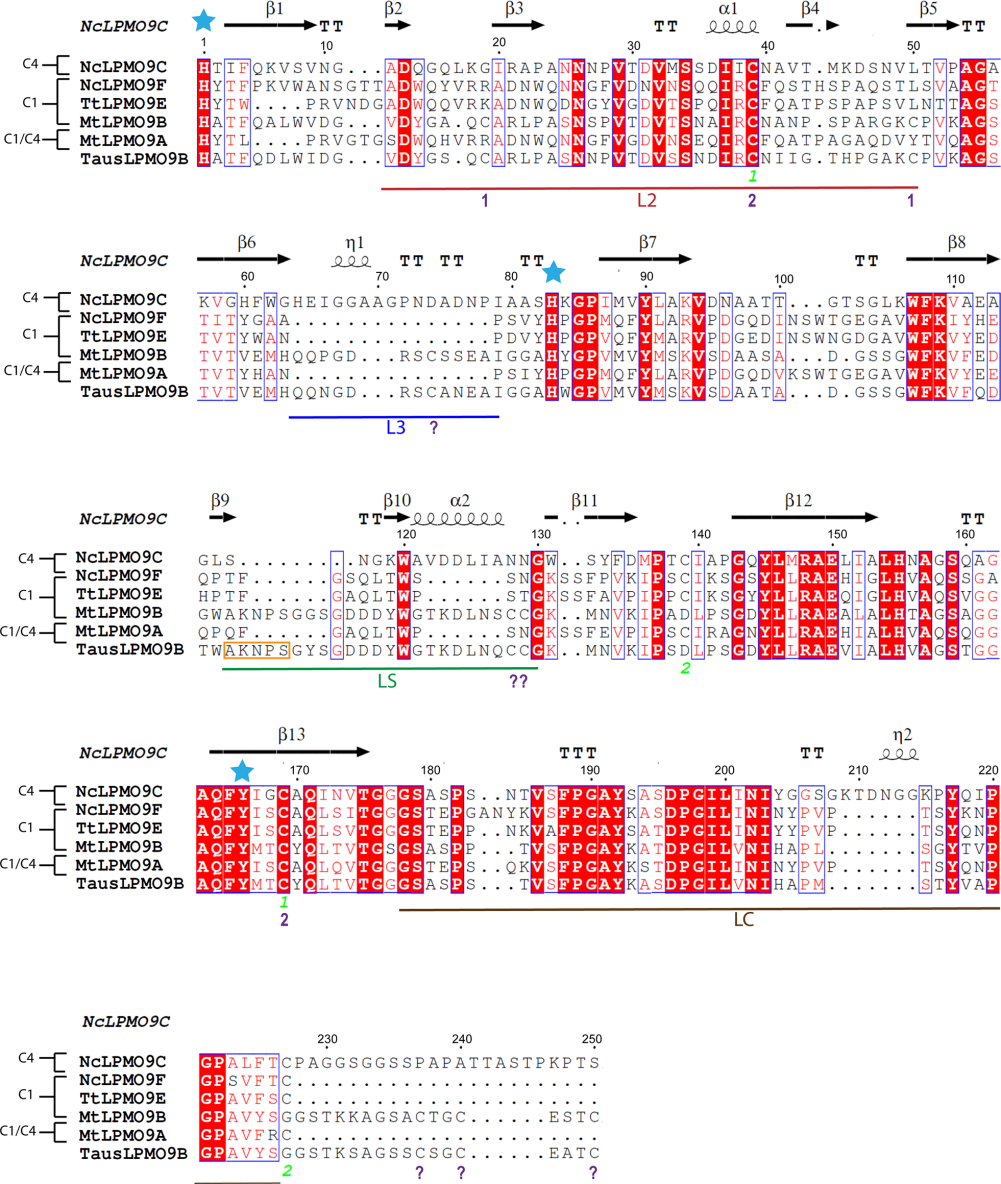
Amino acid sequence alignment of *Taus*LPMO9B and other LPMOs. The LPMOs include: *Mt*LPMO9A (MYCTH_85556; UniProt ID, G2QNT0)*, Mt*LPMO9B (MYCTH_80312; UniProt ID, G2QCJ3), *Tt*LPMO9E (PDB ID, 3EJA; Uniprot ID, D0VWZ9), *Nc*LPMO9C (PDB ID, 4D7U; Uniprot ID, Q7SHI8) and *Nc*LPMO9F (PDB ID, 4QI8; Uniprot ID, Q1K4Q1). Fully conserved residues appear as white letters on red background. Residues that are highly conserved and share similar physico-chemical properties appear as red letters in blue squares. Residues involved in shaping the copper site, including the copper-binding histidine brace, are marked with blue asterisks. The secondary structure features indicated above the sequences are based on the *Nc*LPMO9C structure [69] [β-strands, arrows; α-helices, coils; turns, T]. The residue numbering applies to *Nc*LPMO9C. Previously annotated regions of high sequence variability and with a dominating loop structure, named L2, L3, LS and LC [75], are indicated by red, blue, green and brown bars, respectively, below the sequences. The experimentally determined oxidative regioselectivities of the LPMOs (C1, C4, C1/C4) are indicated to the left. Digits and question marks below the sequence indicate cysteine residues involved in the formation of disulfide bridges; green digits apply to *Nc*LPMO9C, purple digits mark the ones predicted for *Taus*LPMO9B and purple question marks indicate additional cysteines in *Taus*LPMO9B. ESPript was used to generate the figure [67]. The predicted N-glycosylation site is marked in an orange box

cDNA from *T. australiensis* encoding an AA9 LPMO (Table S1) was cloned in *A. niger* [34]. The enzyme was expressed and active, making this LPMO a suitable candidate for biochemical and functional characterization. The product encoded by the 984-basepair-long gene was named *Taus*LPMO9B (due to its high sequence identity with *Mt*LPMO9B from *M. thermophila*; Additional file 1: Table S2) and consists of a typical N-terminal AA9 domain, a linker region and a C-terminal CBM1 domain (Additional file 1: Table S1). Figure 1 shows a multiple sequence alignment of the catalytic domains of *Taus*LPMO9B and selected LPMOs with experimentally confirmed oxidative regioselectivity (i.e. strict C1-, strict C4- or mixed C1/C4-oxidizing LPMOs) from *M. thermophila* (*Mt*LPMO9B, UniProt ID G2QCJ3, strictly C1-oxidizing with 84.8% sequence identity; *Mt*LPMO9A, UniProt ID KP901251, C1/C4-oxidizing with 44.7% sequence identity), *Neurospora crassa* (*Nc*LPMO9F, UniProt ID Q1K4Q1, PDB ID 4QI8, strictly C1-oxidizing with 39.4% sequence identity; *Nc*LPMO9C, UniProt ID Q7SHI8, PDB ID 4D7U, strictly C4-oxidizing with 47.2% sequence identity), and *T. terrestris* (*Tt*LPMO9E, UniProt ID G2RGE5, PDB 3EJA, strictly C1-oxidizing with 43.9% sequence identity). The alignment (Fig. 1) and a structural model built using the structure of *Nc*LPMO9C (PDB ID, 4D7U; Additional file 1, Fig. S1) show that the copper ion is coordinated by the fully conserved His1 and His79, whereas the fully conserved Tyr170 (at position 166 in Fig. 1) is also important for shaping the copper-binding site. Similarly to *Nc*LPMO9C and *Mt*LPMO9B, *Taus*LPMO9B shows an insertion in the L3 region. The enzyme is predicted to have only one *N*-glycosylation site (Asn113) and 24 putative O-glycosylation sites, which are mostly located in the linker and CBM region.

Intriguingly, the complete amino acid sequence of *Taus*LPMO9B contains 16 cysteines, an unusually high number, of which seven are predicted to be located in the AA9 catalytic domain (Cys18, 38, 49, 70, 132, 133 and 173), three are predicted to be part of the linker region adjacent to the AA9 domain (Cys233, 236 and 240) and six are present in the CBM1 module (Cys271, 279, 287, 290, 296 and 307) (Additional file 1, Table S1). The six Cys residues in the CBM1 module will expectedly form three disulfide bonds (Cys271–Cys287, Cys279–Cys296 and Cys290–Cys307) in the CBM1 domain as found in the CBM1 of the endoglucanase *Hj*Cel7B from *H. jecorina* (PDB ID, 4BMF). Notably, all ten Cys residues in the predicted AA9 and linker regions are conserved among a small group of AA9 LPMOs that include *Taus*LPMO9B, the LPMOs listed in Table S2 in Additional file 1 (with 71–83% identity; including *Mt*LPMO9B) as well as *Nc*LPMO9E (UniProt ID, Q7RWN7) and *Nc*LPMO9J (UniProt ID, Q7SHD9) from *N. crassa* and *Pa*LPMO9B (UniProt ID, B2AVF1) from *Podospora anserina* (with 68–75% identity). While regioselectivity has been reported for several of these LPMOs, protein structure data are only available for LPMOs with low (< 50%) sequence identity that do not contain such a high number of Cys residues. (*Nc*LPMO9C shares the highest, but only 47.2%, sequence identity with *Taus*LPMO9B and contains four Cys residues in the AA9 domain, two of which do not occur in *Taus*LPMO9B.) Even in the absence of a protein structure, the fact that the three Cys residues located at the N-terminal end of the linker region (within the first 17 amino acids) are conserved among this group of LPMOs strongly suggests that this part of the linker may have a strong interaction with the AA9 domain, likely forming disulfide bridges. In this respect, it is noteworthy that X-ray crystallographic studies have revealed close interactions between the N-terminal, cysteine-free, part of the linker region of *Hj*LPMO9A from *Hypocrea jecorina* (PDB ID, 5O2W) and its catalytic domain [43]. A closer look at the sequence alignment (Fig. 1 herein and Supplementary Figure S1 in reference [44]) reveals that the Cys39–Cys169 pair (Cys38–Cys173 in *Taus*LPMO9B) comprises the only two Cys residues that are conserved in all AA9 LPMOs. In some AA9 LPMOs, including *Taus*LPMO9B and *Mt*LPMO9B, the L2 loop contains two Cys residues that are positioned such as to form a disulfide bond (positions 19 and 50 in Fig. 1; Cys18 and Cys49, respectively, in *Taus*LPMO9B). The bonding status of the remaining three cysteines in *Taus*LPMO9B, Cys70, Cys132 and Cys133 (positions 74, 128 and 129 in Fig. 1) is not known. It is worth noting that these positions are all surface located and that this is an odd number of cysteines, which may be taken to support the idea that the N-terminal part of the linker, which also contains an odd number (three) of cysteines, interacts with the catalytic domain. Laurent et al. have suggested that the cysteines at positions 74 and 129 form a disulfide bridge in *Mt*LPMO9B, which could pull the L3 loop away from the substrate-binding surface, perhaps creating a surface structure similar to that in (C1-oxidizing) LPMOs lacking the L3 insertion altogether [44].

### Protein expression, purification and mass spectrometry analysis

*Taus*LPMO9B was recombinantly produced in *A. niger*, with a yield of approximately 0.1 g protein per L culture. The protein was purified in a two-step protocol until about 95% purity was obtained (Additional file 1: Fig. S2). A peptide map of the primary sequence was made after tryptic digestion, using LC–MS/MS. The peptide map covered ~ 70% of the amino acid sequence and showed methylation of the N-terminal peptide (Additional file 1: Table S3). The N-terminal peptide, residues 1–20, was detected in both the methylated and non-methylated forms, and the data indicated that 91.7% of the N-terminus was methylated (Additional file 1: Fig. S3). The immonium ion of the methylated histidine was detected in the fragmentation spectrum of the methylated N-terminal peptide (Additional file 1: Fig. S4), confirming that the methylation occurred on the His1 and not on another amino acid in the N-terminal peptide. The peptide containing the potential N-glycosylation site (residues 113–127) was only detected in a form without attached sugars. Also, a parallel analysis of the tryptic digest of the protein after deglycosylation using PNGase F and Endo Hf showed no evidence of glycosylation on Asn113 (data not shown).

A profile of the mature proteoforms originating from varying covalent modifications was obtained by LC–MS analysis of the intact protein. For the deconvoluted MS spectrum of the main chromatographic peak, see Fig. S5 in Additional file 1. The theoretical molecular mass of the mature protein without any PTMs, but lacking the signal peptide, was predicted to be 31,355 Da. The potential PTMs include formation of disulfide bridges (Δ*m/z* = − 2; 16 cysteines in total, so maximally − 16), methylation of the N-terminal histidine (Δ*m/z* =  + 14) and *O*-glycosylation (Δ*m/z* =  + 162 per hexose unit). The deconvoluted mass spectrum of the main chromatographic peak (Additional file 1: Fig. S5) shows a distribution of proteoforms with mass increments of Δ*m/z* = 162.14, indicative of heterogenous glycosylation reflected in a varying number of attached hexoses. The lowest, clearly distinguishable, mass in the deconvoluted mass spectrum was *m/z* = 33,136 [M + H^+^], indicating heavy *O*-glycosylation of *Taus*LPMO9B. The observed difference between the expected (*m/z* = 31,356 [M + H^+^]) and observed masses (from *m/z* = 33,136 to *m/z* = 37,677 [M + H^+^] with mass increments of Δ*m/z* = 162; Fig. S5 in Additional file 1) suggests a range of 11–39 hexoses on the most abundant proteoforms of *Taus*LPMO9B.

### Substrate specificity and regioselectivity on cellulose

**Fig. 2:**
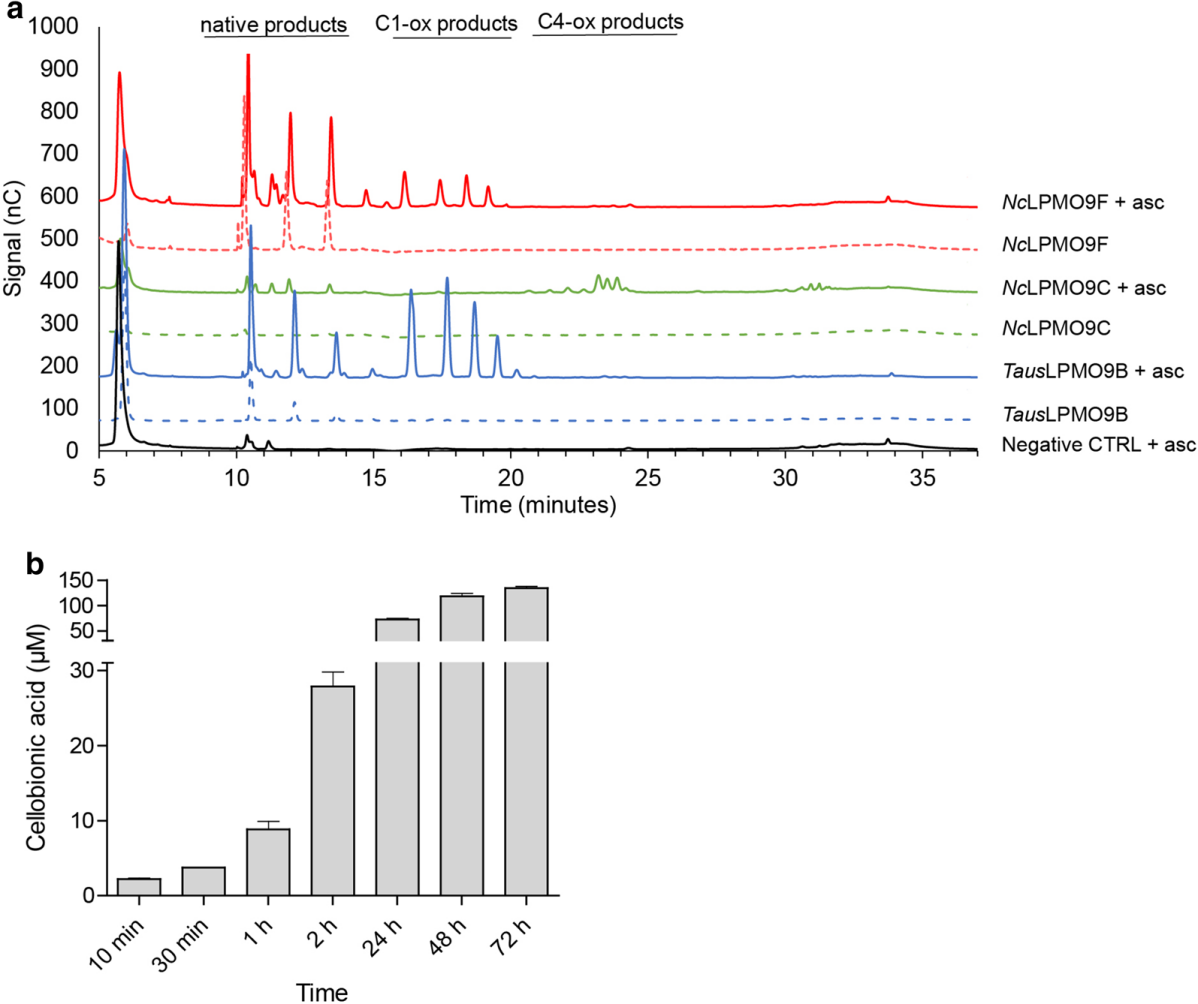
Activity of *Taus*LPMO9B on PASC*.* **a** HPAEC–PAD chromatogram for products released after 24 h incubation at 45 °C, pH 5.0, in the presence (continuous line) or absence (dotted line) of 1 mM ascorbic acid (*Taus*LPMO9B chromatograms are shown in blue, *Nc*LPMO9C in green, *Nc*LPMO9F in red, and the negative control in black). The reactions contained 0.1% (w/v) PASC and1 µM enzyme. **b** Accumulation of cellobionic acid over time in a reaction with 1 µM *Taus*LPMO9B, 0.1% (w/v) PASC and 1 mM ascorbic acid. Cellobionic acid release by *Taus*LPMO9B was quantified by intrapolation of the integrated peak area of the C1-oxidized dimer with a standard curve of cellobionic acid standard solutions (10–50 µM). (Note that cellobionic acid corresponds to only a fraction of the LPMO products as longer C1-oxidized cello-oligosaccharides were not converted to dimers before HPAEC–PAD analysis)

*Taus*LPMO9B was incubated with PASC to monitor its activity over time and determine its regioselectivity, in the presence of ascorbic acid as electron donor. As positive controls, C1-oxidizing *Nc*LPMO9F and C4-oxidizing *Nc*LPMO9C [45] were incubated under the same conditions. C4-oxidized products could be detected for the reactions driven by *Nc*LPMO9C, while *Nc*LPMO9F and *Taus*LPMO9B only released C1-oxidized products (Fig. 2a). Monitoring of the accumulation of oxidized products over time showed that under the conditions tested (pH 5, 45 °C) the enzyme was highly active for at least 24 h (Fig. 2b). While experimental data show that, similarly to its closest homologue *Mt*LPMO9B, *Taus*LPMO9B is a strictly C1-oxidizing LPMO, it is not yet fully clear which structural features determine the stereospecificity of LPMOs [46, 47]. Earlier studies correlated the presence of an insertion in the L3 region with a preferential C4-oxidizing activity [48]. More recently, Laurent et al. showed that this type of insertion, which they refer to as Segment 2, is not always associated with strictly C4-oxidizing activities [44], as is corroborated by the data presented here for *Taus*LPMO9B. A phylogenetic tree analysis revealed that, indeed, *Taus*LPMO9B clustered with other C1-oxidizing LPMOs (Fig. S6 in Additional file 1). This confirms other recent studies showing that identifying local pairwise alignments in the phylogenetic tree is a better approach, as it is not yet clear which are the structural features determining LPMOs’ stereospecificity [49]. Of note, there are more examples of discrepancies between predicted and observed regioselectivities. In 2015, Bennati et al. showed that *Pa*LPMO9H, predicted to be a C4-oxidizing LPMO, was actually producing C1- and C4-oxidized products [50]. Before this, biochemical characterization of *Mt*PMO3* (MYCTH_92668) resulted in its classification as a C1-oxidizing LPMO despite its predicted C1/C4-oxidizing regioselectivity [48]. Taken together, these examples highlight that more biochemical and structural data are needed to determine, predict and understand the structural determinants of regioselectivity.

Similarly to *Mt*LPMO9B [19], no oxidized products were detected upon incubation with hemicellulosic substrates, such as β-glucan, glucomannan, arabinoxylan, xyloglucan, xylan (alone and in combination with PASC; Additional File 1: Fig. S7), and soluble oligosaccharides, such as cellopentaose.

**Fig. 3:**
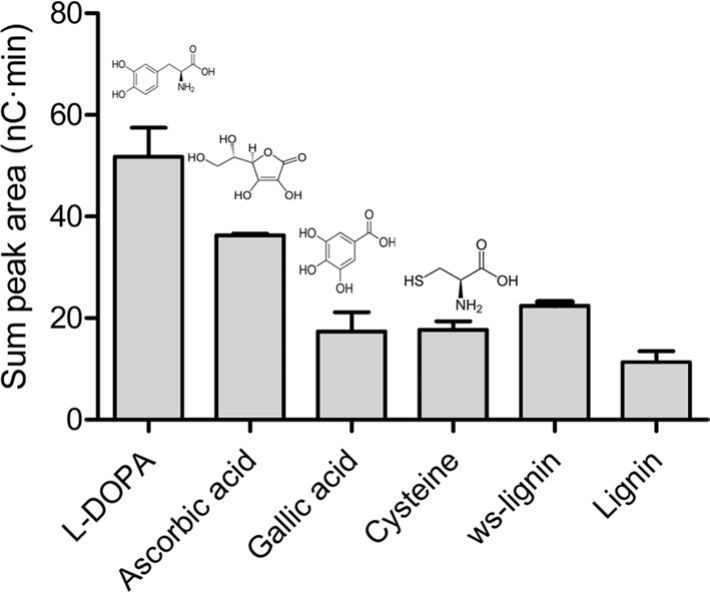
Activity of *Taus*LPMO9B in the presence of various reductants. The graph shows the release of oxidized products by *Taus*LPMO9B in reactions with different reductants. The chemical structures of the reductants are indicated above the bars. Reactions contained 1 µM LPMO, 0.1% (w/v) PASC and either 1 mM of small reducing compounds, 1 mg/mL of kraft lignin or 1% (v/v) of water-soluble lignin (ws-lignin). Product release was measured after 24-h incubation at 45 °C and pH 5.0 and is reported as the sum of the area of peaks corresponding to C1-oxidized cello-oligosaccharides (DP2–DP6). Due to the high background signal of the extracted lignin (ws-lignin) overlapping with signals of the LPMO products, the results for this set of experiments were background corrected. In the control sample, PASC was incubated with ws-lignin but without enzymes, using the same reaction conditions as in the reaction containing the enzyme. Reactions were run in triplicate, error bars indicate standard deviation

The presence of reductant is key for the LPMO reaction, and the efficiency of different reductants to drive the reaction varies between LPMOs [19]. Differences are caused by many aspects, including the reactivity of the reductant with O_2_ and dependency on pH [51, 52]. As for reactions with biomass, it is well known that lignin and/or lignin-derived compounds may act as reductant and that addition of an external reductant may be needed in cases where the pretreatment of biomass drastically decreases the lignin content [10, 21, 53, 54]. Frommhagen et al. determined reductant preferences for *Mt*LPMO9B from *M. thermophila*, the AA9 domain of which shares 83.9% identity with *Taus*LPMO9B [19]. Inspired by this previous work, we tested compounds containing either sulfur (l-cysteine), a benzenediol moiety (L-DOPA) or a benzenetriol moiety (gallic acid), since these all had a positive effect on *Mt*LPMO9B [19]. In addition, commercial lignin, as well as the water-soluble lignin extracted from acid pretreated corn stover, were tested. Similarly to what was observed for *Mt*LPMO9B, L-DOPA and ascorbic acid were the most efficient reductants driving *Taus*LPMO9B activity, leading to the release of a higher amount of oxidized products after 24 h (Fig. 3).

### Thermal stability and pH dependency of TausLPMO9B

**Fig. 4:**
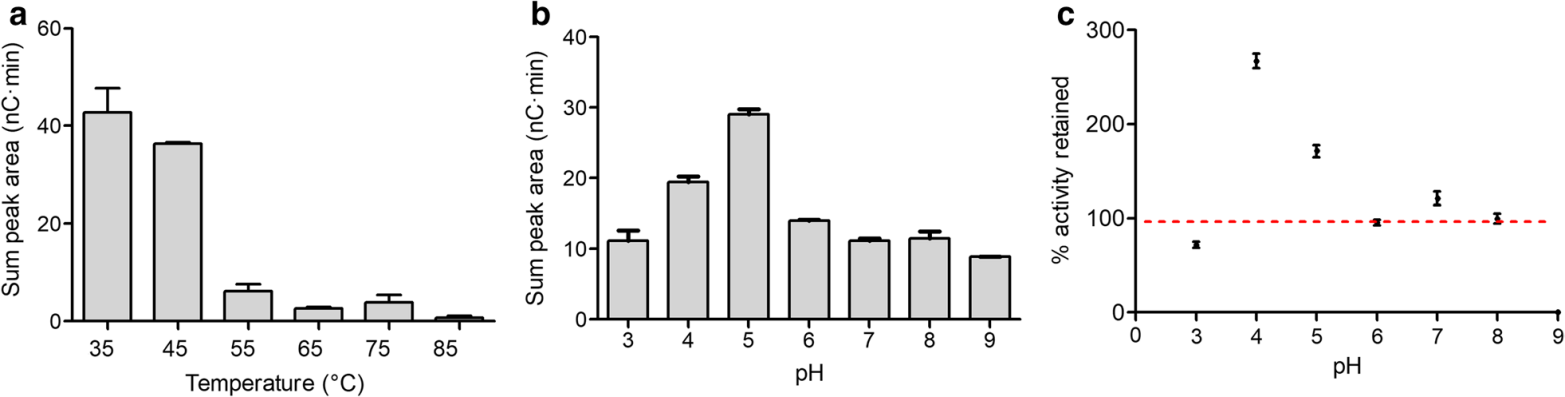
Effect of temperature and pH on *Taus*LPMO9B. Reaction mixtures containing 1-µM LPMO, 1-mM ascorbic acid and 0.1% w/v PASC were incubated for 24 h at different conditions: **a** fixed pH 5.0, different temperatures (35 °C, 45 °C, 55 °C, 65 °C, 75 °C and 85 °C); **b** fixed temperature 45 °C, different pHs (3.0, 4.0, 5.0, 6.0, 7.0, 8.0 and 9.0). The amount of total oxidized products was calculated as sum of the peak areas of peaks eluting between 16 and 30 min during HPAEC–PAD (i.e., C1-oxidized cello-oligosaccharides, DP2 – DP6). The stability of *Taus*LPMO9B in the function of pH (**c**) was evaluated by pre-mixing the enzyme with buffers of varying pH in 50% of the final reaction volume and incubating for 24 h at 45 °C, after which PASC (0.1% w/v) and ascorbic acid (1 mM) were added to initiate the reaction, followed by incubation for 24 h at 45 °C. Control reactions at the same varying pH values but without the pre-incubation step were also set up. The data points represent the percentage of activity retained by the pre-incubated enzyme, relative to the non-pre-incubated enzyme. Activities were calculated as the sum of peak areas corresponding to oxidized products in the HPAEC–PAD analysis. In other words, 100% retained activity (marked by a red line) means that the amount of oxidized products released was identical in the reactions with and without pre-incubation

The effects of pH and temperature on the activity of *Taus*LPMO9B were investigated using PASC as substrate. Under the conditions tested, *Taus*LPMO9B released the highest amount of oxidized products in the range of 35–45 °C (Fig. 4a). The apparent enzyme activity was drastically reduced at higher temperatures (Fig. 4a), which was surprising considering the fact that protein stability measurements with the Thermofluor assay (Table 1 and Fig. S8 in Additional file 1) indicated an apparent melting temperature around 75 °C. It is likely that under the conditions used here, the thermal stability of the reducing agent ascorbic acid and/or limited solubility of oxygen at increased temperatures [19, 51, 52, 55] may have come into play. Almost no products were detected after 24 h of incubation at 85 °C.

**Table 1:** The effect of pH on the apparent melting temperature (*T*_m_) of TausLPMO9B

pH	Melting temperature (°C)
3	61.2 ± 0.7
5	76.3 ± 0.3
7	75.8 ± 0.5
8	74.5 ± 0.4

Next, we assessed the effect of pH on *Taus*LPMO9B activity. It should be noted that the pH has an indirect impact on LPMO product formation due to the pH dependency of the reducing capacity of the reducing agent (i.e., ascorbic acid) [51, 52]. Under the conditions used here, the enzyme released most oxidized products at a pH between 4 and 6, with the highest level detected at pH 5 (Fig. 4b). As most of the industrial cellulolytic enzymes have an optimum around pH 5 [56], this makes *Taus*LPMO9B, under the tested conditions, highly suitable to complement such enzymes. To assess the stability of *Taus*LPMO9B at different pH conditions, the enzyme was pre-incubated in buffer at a pH ranging from 3.0 to 8.0 for 24 h at 45 °C. Comparison of the initial and residual activities of the enzyme, based on the formation of oxidized products released from PASC in a 24-h reaction at 45 °C at the pH of the pre-incubation (Fig. 4c), revealed some loss of activity at pH 3. Protein stability measurements using the Thermofluor assay confirmed that *Taus*LPMO9B was significantly less stable at pH 3.0 (apparent *T*_m_ of 61 °C at pH 3.0) than at pH 5.0–8.0 (apparent *T*_m_ around 75 °C) (Table 1 and Fig. S8 in Additional file 1).

Melting temperatures were measured at different pH values using the Thermofluor assay, as described in the legend of Fig. S8 in Additional file 1, which shows raw data. The analyses were run in triplicates; errors correspond to standard deviations.

While *Taus*LPMO9B proved to be a rather stable enzyme at all tested pH values, we observed an intriguing increase in LPMO activity upon overnight pre-incubation of the enzyme at pH 4.0 and 5.0 (Fig. 4c), for which we currently do not have an explanation. It is clear though from the above observation that optimal conditions for studying the biochemical properties and industrial applicability of *Taus*LPMO9B entail a pH of around 5 and a temperature of 45 °C.

### *The role of H*_*2*_*O*_*2*_* as a co-substrate*

**Fig. 5:**
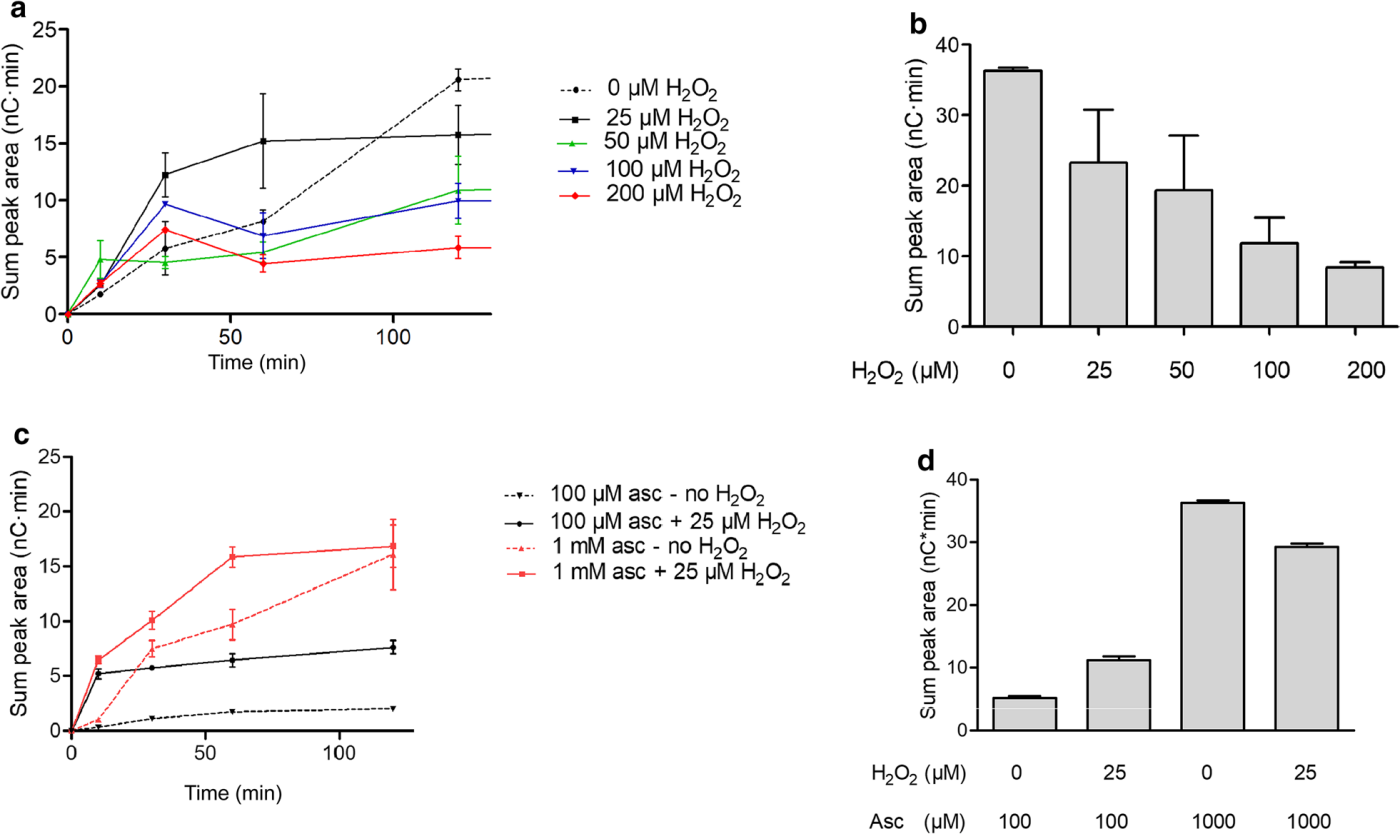
Effect of H_2_O_2_ and ascorbic acid on *Taus*LPMO9B activity on cellulose. 0.1% (w/v) PASC was incubated with 1-µM *Taus*LPMO9B acid in sodium acetate buffer pH 5.0 at 45 °C with different combinations of reductant/H_2_O_2_. **a**, **b**: 1-mM ascorbic acid and supplementation of different concentrations of H_2_O_2_ (0-200 µM). At time ‘0’, the reactions were supplemented with 0–200 µM H_2_O_2_. Product formation was assessed based on the sum of peak areas corresponding to C1-oxidized cello-oligosaccharides in the HPAEC–PAD chromatograms. **c**, **d** 0.1% (w/v) PASC was incubated with 1 µM *Taus*LPMO9B and either 100-µM or 1-mM ascorbic acid in sodium acetate buffer pH 5.0 at 45 °C. At time ‘0’, the reactions were supplemented with 0 (ultra pure water) or 25-µM H_2_O_2_. Product formation was assessed based on the sum of peak areas corresponding to C1-oxidized cello-oligosaccharides in the HPAEC–PAD chromatograms. **a**, **c** Product accumulation in the first 120 min, the lines connecting the points are drawn for illustration purposes only; **b, d** final product levels after 24 h. The error bars indicate standard deviations of three replicates

As recent studies reported that LPMOs are more efficient when fueled with H_*2*_O_2_ as co-substrate instead of O_2_ [18, 28, 57, 58], we assessed the suitability of H_2_O_2_ as co-substrate for *Taus*LPMO9B. First, PASC was incubated with *Taus*LPMO9B, H_2_O_2_ and an excess of reductant (1-mM ascorbic acid). Under the studied conditions, supplementing the reaction with H_2_O_2_ (at 25–200 µM concentration) had a positive impact on the initial rate of product formation, while higher concentrations of H_2_O_2_ (here 100–200 µM) were detrimental for the enzyme and led to an early stop of product accumulation (Fig. 5a). Our results are in agreement with previous studies showing that low concentrations of H_2_O_2_ are able to boost LPMO activity, while an excess of H_2_O_2_ leads to inactivation of the enzyme, presumably through oxidative damage, due to the fact that the non-substrate-bound fraction of reduced LPMOs will react with H_2_O_2_ and catalyze self-inactivation [18]. Figure 5b shows a negative correlation between the amount of H_2_O_2_ supplied to the reaction and product levels obtained after 24-h incubation; reactions with added H_2_O_2_ yielded less oxidized cello-oligosaccharides than those where H_2_O_2_ was not added. At this high ascorbic acid concentration and in the absence of added H_2_O_2_, the reaction may be slower but proceeds for a much longer time, leading to the highest product levels. In the reactions with added H_2_O_2_, multiple deleterious effects may occur, including direct autocatalytic inactivation of the enzyme, as mentioned above, and reactions between the reductant and H_2_O_2_, which reduces the concentration of both and may generate potentially damaging reactive oxygen species.

To further elucidate the role of H_2_O_2_ in the action of *Taus*LPMO9B, we reduced the concentration of ascorbic acid in the reaction to 100 µM, which is in line with the hypothesis that, to perform their oxidative reaction using H_2_O_2_, LPMOs would only require low amounts of reductant [18]. In this setup, the effect of adding H_2_O_2_ was much more pronounced. Adding only 25-µM H_2_O_2_ to the reaction mixture with 100-µM ascorbic acid led to a 16-fold increase in product formation after 10 min (Fig. 5c) and a twofold increase in the overall product yield after 24 h (Fig. 5d). Under these conditions, there is ~ 190-µM O_2_ present [55] and 25-µM H_2_O_2_, suggesting that the affinity of *Taus*LPMO9B for H_2_O_2_ is higher than for O_2_, leading to a faster initial activity. These findings corroborate earlier studies showing that H_2_O_2_ is utilized preferentially by LPMOs compared to O_2_ [18, 28]. The best performance over 24 h was achieved without H_2_O_2_ addition, using higher amounts of reductant in the presence of oxygen (Fig. 5d). A plausible explanation for this could be that, in the presence of ascorbic acid and molecular O_2_, H_2_O_2_ is formed slowly and steadily, driving the enzyme reaction [18]. In this manner, H_2_O_2_ concentrations would never become high (likely never above a few micromolar [28, 54]), which avoids enzyme damage. These observations suggest that it would be better to drive the enzyme reaction with the help of an enzymatic redox partner producing H_2_O_2_ in situ, such as cellobiose dehydrogenase [59], rather than with externally supplied H_2_O_2_. Such enzymes are abundant in fungal secretomes [58].

### Hydrolysis of pretreated corn stover

**Fig. 6:**
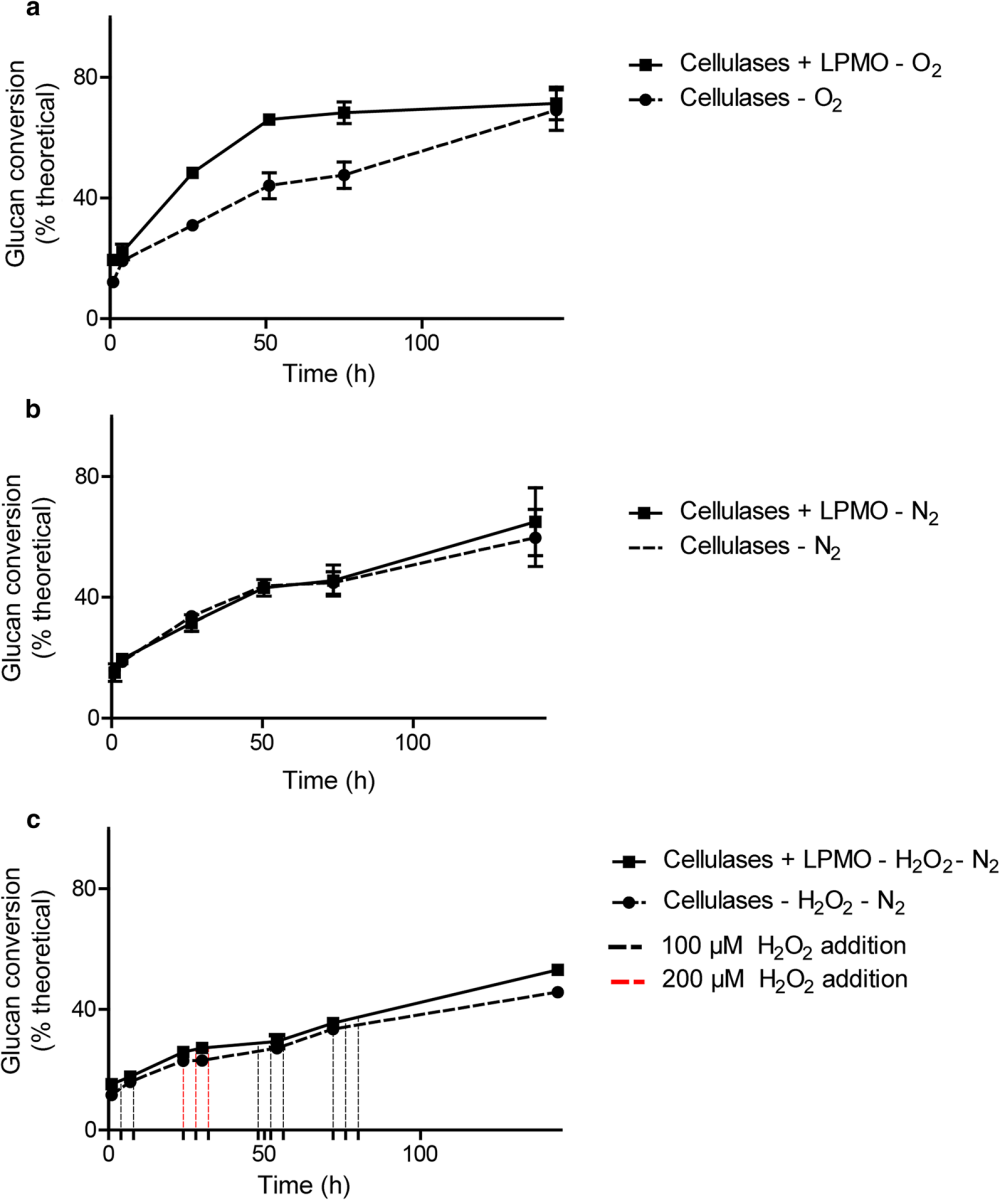
Effect of *Taus*LPMO9B in hydrolysis of acid pretreated corn stover (PCS). PCS at 10% DM (w/v) was incubated in bioreactors with a mixture of Celluclast^®^-BG, with (continuous line) or without (dotted line) additional LPMO under different conditions: **a** constant sparging of headspace with air, **b** constant sparging with N_2_, and **c** constant sparging with N_2_, with the addition of H_2_O_2_ three times a day to a final concentration of 100 µM (black dotted lines) or 200 µM (red dotted lines) per addition. The dosage was increased as no clear effect was detectable on the glucose release levels after the first three dosages. Samples were collected at different time points and enzymes were immediately inactivated by addition of 4-N NaOH. Glucose was quantified by HPLC. The error bars indicate standard deviations of two replicates

To examine the potential of *Taus*LPMO9B to enhance the performance of classic hydrolytic cellulase cocktails, like Celluclast^®^, during the saccharification of industrially relevant lignocellulosic material using oxygen (supplied as air) or H_2_O_2_ as co-substrate, hydrolysis experiments with acid pretreated corn stover were performed. To simulate industrial processing, reactions were carried out in 1.5-L fermenters where the composition of the headspace could be controlled to ensure aerobic or anaerobic conditions and the stirring power allowed proper mixing of 10% (w/w) dry matter slurries. The boosting effect of *Taus*LPMO9B was tested via spiking a cellulase cocktail (BG-enriched Celluclast^®^) with the LPMO both in the presence and absence of oxygen (supplied as air) or H_2_O_2_. Based on previous studies using different dosages of H_2_O_2_ [18, 54] and taking into consideration possible side reactions of H_2_O_2_ with feedstock components [23], it was decided to spike 100–200 µM H_2_O_2_ three times per day to the bioreactors, to ensure sufficient supply of hydrogen peroxide. In total, six bioreactors were set up: three containing BG-enriched Celluclast® and three containing BG-enriched Celluclast^®^ spiked with LPMO. The saccharification reactions with and without LPMO were carried out under aerobic conditions (oxygen supplied via headspace sparging with air) (Fig. 6a) and anaerobic conditions (headspace sparging with N_2_), the latter of which was without or with spiking of H_2_O_2_ (Fig. 6b, c, respectively).

The results showed that addition of *Taus*LPMO9B was able to improve the initial rate of glucan conversion by the Celluclast^®^/BG mixture in the presence of oxygen (Fig. 6a). This is in agreement with earlier observations showing the ability of LPMO to boost the conversion of lignin-containing feedstocks in the presence of oxygen [21, 53, 54]. As expected, the LPMO effect could not be detected in saccharification reactions operated under anaerobic conditions (Fig. 6b). Furthermore, in contrast to the assays performed on PASC at small scale without pH and aeration controls (see Fig. 5), addition of H_2_O_2_ had no significant effect on the outcome of the saccharification reaction, indicating that under these anaerobic conditions, the LPMO stayed inactive (Fig. 6c). We propose that the lack of the effect of H_2_O_2_ is due to the presence of lignin, which may react with H_2_O_2_ before the LPMO can use it as a co-substrate.

So far, only a handful of studies have been conducted to address the effect of H_2_O_2_ additions on the efficiency of the saccharification of different types of biomass with LPMO-containing enzyme cocktails [18, 54]. Comparing the saccharification of Avicel and two types of industrially relevant biomasses (sulfite-pulped Norway spruce and steam-exploded birch), the seminal study by Müller et al. [54] showed that the presence of lignin in the biomass decreased the efficiency with which the LPMO was able to use externally supplied H_2_O_2_. In agreement with this, a recent study by Kont et al. [23] has shown that lignin has both H_2_O_2_-forming and -scavenging activities and suggested that H_2_O_2_ is an intermediate of lignin oxidation. Hence, it is plausible that lignin reacts with the supplied H_2_O_2_ before it reaches the LPMO. Potentially, this could lead to generation of reactive oxygen species that may contribute to inactivation of LPMOs and other enzymes in the reaction. While there are several indications in literature that lignin is important for driving LPMO action on industrial substrates in the presence of O_2_ [20, 24, 53], the type of substrate, its pretreatment and, hence, its composition will affect the ability of LPMOs to use externally supplied H_2_O_2_ as a co-substrate (this study; [54]).

## Discussion

The number of LPMOs in (thermophilic) filamentous fungi and their expression levels vary to a great extent. The genome of *T. terrestris*, for example, encodes nearly 20 AA9 LPMOs [60]. *T. aurantiacus*, on the other hand, encodes only a handful of AA9 LPMOs in their genome [61]. Here, we report the characterization of *Taus*LPMO9B from the thermophilic fungus *T. australiensis* and describe its potential to use O_2_ and H_2_O_2_ as co-substrate in reactions on both model and industrial substrates. *Taus*LPMO9B was found stable at slightly acidic pH and active on lignocellulosic feedstocks, such as acid pretreated corn stover, indicative of its potential suitability for supplementing today’s commercial cellulase cocktails for biomass saccharification. The efficiency of the enzyme was dependent on the type of cellulosic substrates; on “clean” model celluloses, addition of H_2_O_2_ promoted faster initial activity and, in some cases, a higher final product yield; whilst on industrially relevant lignocellulosic materials, reactions running in the presence of O_2_ only led to a faster overall saccharification. As the lignin may react with the added H_2_O_2_ [23] and, thus, limits the availability of H_2_O_2_ for the LPMO, it would be interesting to investigate other options for its supply. An interesting option would be the use of auxiliary enzymes, such as cellobiose dehydrogenases (CDH). Kracher et al. have demonstrated that LPMO reactions fueled by an engineered CDH with increased oxidase activity are faster and more steady, due to a higher H_2_O_2_ production rate compared to the wild-type enzyme [59]. It might be useful to use both LPMOs and H_2_O_2_-generating enzymes that contain CBMs, since this could assure that H_2_O_2_ is being generated close to the substrate and the LPMO, which could reduce side reactions of H_2_O_2_ with lignin [62].

For the characterization of the properties of *Taus*LPMO9B, commonly applied reaction setups were used wherein the reaction is driven by oxidation of the reductant, in most cases ascorbic acid, either by direct reactions with O_2_, or by reactions involving the LPMO. If one accepts the increasingly supported notion that H_2_O_2_ is a kinetically more relevant co-substrate of LPMOs [18, 28, 31], the reactions carried out here (and in many previous studies) may be limited by in situ generation of H_2_O_2_ rather than by the catalytic ability or stability of the LPMO. Observed differences in reductant efficiency could relate to the generation of hydrogen peroxide rather than to the ability to reduce the LPMO. Moreover, factors such as pH and temperature will affect important reaction parameters next to the catalytic activity of the LPMO, including the level of dissolved oxygen and the oxidative stability of the reductant, which, together, will affect the formation of H_2_O_2_. Therefore, it cannot be excluded that reactions driven by gradual supply of appropriate amounts of H_2_O_2_ would yield other pH or temperature optima, or even substrate specificities, although existing data indicate that the latter does not vary between O_2_- and H_2_O_2_-driven reactions [33].

## Conclusions

Since the discovery of their ability to boost saccharification of lignocellulosic material only a decade ago, LPMOs have been the object of intense research to harvest their full potential in enzymatic saccharification. Unfortunately, in industrially relevant settings (i.e., 15–20% w/w pretreated biomass, containing much more than only cellulose), assessing LPMO activity is rather challenging because a high background from compounds present in the pretreated feedstock makes detection of LPMO-derived oxidized reaction products challenging. Detection and identification of LPMO products require, most of the time, the use of “clean” model substrates such as Avicel or PASC. It has previously been shown that the observed activity of LPMOs on “real” feedstocks differs from the results obtained with model substrates [54]. It has also been shown that harnessing the full potential of the enzyme requires a delicate balancing act between activation, the presence of co-substrates (O_2_ or H_2_O_2_) and inactivation [18, 28].

Our present findings corroborate the notion that the current parameters used in industrial-scale saccharification remain to be optimized to leverage LPMO performance on lignin-rich substrates to the same extent as what can be achieved with “cleaner” substrates. Since the impact of LPMOs on biomass saccharification is considerable and indisputable, further research on the redox processes that happen in reactions with real biomass is of major interest.

## Methods

### Production and purification of enzymes

The gene encoding for *Taus*LPMO9B was PCR amplified from a cDNA library of *T. australiensis* strain ATCC 28,236 and expressed in *A. niger.* In short, the *Taus*LPMO9B gene (sequence ID number 1 in reference 34) was PCR amplified using the forward primer 5′-CCCCAGCAACAAAACACCTCAGCAATGAAGTCGTTCACCGTTGCC-3′, the reverse primer 5′-GAAGGACGGCGACGGACTTCAGATACACTGGTAGTAGTAAAGG-3′ and a proofreading DNA polymerase (Phusion DNA Polymerase) according to the suppliers’ instructions (Finnzymes). Also, the expression vector pGBFIN-49 [63] was PCR amplified using the forward primer 5′-GTCCGTCGCCGTCCTTCACCG-3′ and the reverse primer 5′-GGTGTTTTGTTGCTGGGGATGAAGC-3′. After ligation-independent cloning (LIC) according to Aslanidis et al. [64], *Taus*LPMO9B was cloned in between the *A. niger glaA* promoter and *trpC* terminator sequences, present on the pGBFIN-49 vector, to drive expression in *A. niger*. The complete expression vector was transfected into *A. niger*, strain GBA307 [63], according to the method described by Punt and van den Hondel [65], and stable phleomycin-resistant transformants were obtained. After 5–10 days, isolated colonies were selected and transferred onto Potato Dextrose Agar (PDA, Difco) supplemented with 150-μg/mL phleomycin. After 5–7 days, spores were harvested by adding sterile water on top of the agar, resuspended and subsequently mixed 1:1 with 50% glycerol for storage at − 80 °C.

For enzyme production, the frozen spore suspension was plated on PDA and incubated at 30 °C. After 7 days, spores were collected by adding sterile water onto the agar, resuspended and washed with sterile water. This spore suspension was used to inoculate a 20-mL pre-culture with corn steep liquor (CSL) medium in a 100-mL baffled flask. CSL medium consisted of (in amount per liter): 100-g Corn Steep Solids (Roquette), 1-g NaH_2_PO_4_·H_2_O, 0.5-g MgSO_4_·7H_2_O, 10-g glucose and 0.25-g Basildon (antifoam). The ingredients were dissolved in demineralized water, the pH was adjusted to pH 5.8 with NaOH or H_2_SO_4_, and the resulting growth medium was sterilized for 20 min at 120 °C. After overnight cultivation in CSL medium at 34 °C and 170 rpm, the 20-mL pre-culture was mixed with 80-mL Fermentation Medium (FM). FM consisted of (in amount per liter): 150-g maltose, 60-g Soytone (peptone), 15-g (NH_4_)_2_SO_4_, 1-g NaH_2_PO_4_·H_2_O, 15-g MgSO_4_·7H_2_O, 0.08-g Tween 80, 0.02-g Basildon (antifoam), 20-g 2-(*N*-morpholino)ethanesulfonic acid (MES) and 1-g l-arginine. The ingredients were dissolved in demineralized water, the pH was adjusted to pH 6.2 with NaOH or H_2_SO_4_, and the resulting fermentation medium was sterilized for 20 min at 120 °C. Growth in FM was performed aerobically in 500-mL baffled flasks with 100-mL final volume at 36 °C and 170 rpm. After 4 days, the broth was collected and centrifuged at 5,000* g* for 10 min to remove cells and debris. The protein content of the supernatant was analyzed by SDS-PAGE (Additional file 1, Fig. S2).

Prior to purification, the supernatant was sterilized by filtration through, first, a 0.45-µm filter and then a 0.22-µm filter, followed by concentration using a Vivaspin 10,000 Da spin column (GE Healthcare). The protein solution was loaded onto a HiPrep 26/10 Desalting Column (Sigma Aldrich) paired with an ÄKTA Prime Chromatography system (7.0-mL/min flow, 50-mM BisTris HCl buffer pH 7.0, 5-mL fractions collected). The elution profile was followed by monitoring the absorbance at 280 and 220 nm. LPMO-containing fractions (based on A_280_ and SDS-PAGE analysis of the collected fractions) were pooled and concentrated by extensive centrifugation in Amicon Ultra centrifugal filters (Millipore) with a molecular mass cut-off of 10,000 Da. Next, the protein was purified further with anion exchange chromatography. A 5-mL HiTrap CaptoQ XL column was equilibrated with 50-mM BisTris HCl buffer (pH 7.0) before loading the protein sample. Elution was performed by applying a linear gradient from 0- to 1-M NaCl in 50-mM BisTris HCl buffer (pH 7.0) at a flow rate of 2 mL/min in 10 column volumes. The elution profile was followed by monitoring the absorbance at 280 and 220 nm. LPMO-containing fractions (based on *A*_280_ and SDS-PAGE analysis of the collected fractions) were pooled and concentrated using Amicon Ultra centrifugal filters with a molecular mass cut-off of 10,000 Da (Millipore). The purity of the resulting *Taus*LPMO9B preparation was confirmed by SDS-PAGE (Additional file 1: Fig. S2). After purification, *Taus*LPMO9B was loaded with copper by incubating with 3-molar-fold excess of CuSO_4_ for 30 min in a thermomixer (Eppendorf ThermoMixer™ C, Hamburg, Germany) at room temperature as described before [66]. Excess copper was removed using a PD10 desalting column containing Sephadex G-25 resin (GE Healthcare Lifesciences), pre-equilibrated with 50-mM sodium acetate buffer pH 5.0. The protein content was then measured using the ThermoScientific Pierce™ BCA assay according to the recommendations of the supplier.

### Structure-based sequence alignment and structural modeling

A comparison between the *Taus*LPMO9B sequence and sequences of other biochemically characterized AA9 was performed using the Clustal Omega multiple sequence alignment program (http://www.ebi.ac.uk/Tools /msa/clust alo/). The structure-based sequence alignment was generated using ESPript [67]. The three-dimensional structure of *Taus*LPMO9B was predicted with the Protein Homology/analogY Recognition Engine V 2.0 [68], and a model was built based on the available structure of the catalytic domain of *Nc*LPMO9C from *N. crassa* [69] (PDB, 4D7U), which shares 44.1% amino acid identity with *Taus*LPMO9B, and visualized with PyMOL version 2.3 (The PyMOL Molecular Graphics System, Version 1.2r3pre, Schrödinger, LLC.). Possible N-and O-glycosylation sites were predicted using NetNGlyc 1.0 (http://www.cbs.dtu.dk/services/NetNGlyc/) and NetOGlyc 4.0 (http://www.cbs.dtu.dk/services/NetOGlyc-4.0/), respectively [70, 71]. Possible disulfide bridges were predicted using DiANNA 1.1 [72] (http://clavius.bc.edu/~clotelab/DiANNA/).

### Screening of substrates and reductants

Unless specified otherwise, reducing agents were purchased from Sigma Aldrich (Steinheim, Germany). Avicel PH-101 was purchased from Merck Millipore (Burlington, MA, USA). β-glucan from oat spelt, glucomannan from konjac, wheat arabinoxylan, xyloglucan from tamarind seeds and cello-oligosaccharides (with a degree of polymerization, DP, of 3–6) were purchased from Megazyme (Bray, Ireland). Xylan from oat spelt was purchased from Serva (Brussels, Belgium). Cellobionic acid sodium salt was purchased from Synthose (Toronto, Canada). Phosphoric acid swollen cellulose (PASC) was prepared from Avicel PH-101 as previously described [73]. In short, 4-mg Avicel PH-101 was suspended in 100-mL *ortho*-phosphoric acid (85% w/v) with constant stirring. Once the cellulose was dissolved, 1900-mL ice cold water was added. The mixture was left to sediment for 1 h at 4 °C. This washing step was repeated three times. Subsequently, the pellet was washed with 1% sodium bicarbonate (pH 7.0) three times, until a neutral pH was reached. The pellet was washed again with water three times, followed by centrifugation at 4000*g* for 10 min. After removal of the supernatant, the pellet was homogenized with a Thorax Blender. The final dry matter content of the PASC was measured with a moisture analyzer (HB43-S Mettler-Toledo, Greifensee, Switzerland).

To test substrate preference, reaction mixtures (250 µL) containing substrate (0.1% w/v), 1-µM LPMO and 1-mM ascorbic acid were incubated in 50-mM sodium acetate buffer pH 5.0 for 24 h at 45 °C, in a thermomixer (Eppendorf ThermoMixer™ C, Hamburg, Germany) with constant shaking at 600 rpm. To stop the reactions, samples were filtered through a 10,000 Da molecular weight cut-off PES filter fitted into an Eppendorf tube at 14,000 rpm for 10 min. As positive controls, reaction mixtures containing 1-µM *Nc*LPMO9C, 1-mg/mL substrate and 1-mM ascorbic acid were prepared.

To test the effect of different electron donors, the same conditions as above were applied using PASC as substrate and either 1-mM L-DOPA, 1-mM cysteine, 1-mM gallic acid, 1-mg/mL kraft lignin (Sigma) or 1% (v/v) water-soluble lignin derived from acid pretreated corn stover. The water-soluble lignin fraction was produced by separating the solid fraction of the pretreated biomass by centrifugation at 4,000* g* for 15 min. Control reactions did not contain any reductant. All reactions were performed in triplicate.

### Enzyme activity

To identify the optimal reaction conditions for *Taus*LPMO9B, different pH values and temperatures were tested. Reaction mixtures (300-µL final volume) containing PASC (0.1% w/v), 1-µM LPMO and 1-mM ascorbic acid were incubated in 50-mM buffer at different pH values (sodium citrate–phosphate for pH 3.0, sodium acetate for pH 4.0 and 5.0, BisTris/HCl for pH 6.0 and 7.0, Tris/HCl for pH 8.0 and 9.0).

To study the activity of *Taus*LPMO9B at different temperatures, the substrate and buffer were pre-incubated at different temperatures (ranging from 35 to 85 °C), before adding the enzyme and reductant to generate reaction mixtures as described above. To follow reactions for longer periods (up to 72 h), the reaction volume was doubled to 600 µL. For sampling, 100 µL was withdrawn and filtered after 10 min, 30 min and 24 h at 4 °C and the solubilized oxidized cello-oligosaccharides in the supernatant were analyzed with high-performance anion exchange chromatography with pulsed amperometric detection (HPAEC–PAD) (see below). All reactions were performed in triplicate.

### *The effect of H*_*2*_*O*_*2*_* on the activity of TausLPMO9B*

To test the potential of H_2_O_2_ as a co-substrate for *Taus*LPMO9B, reaction mixtures (500-µL total volume) containing 0.1% (w/v) PASC, 1-mM or 100-µM ascorbic acid, 1-µM enzyme and various concentrations of H_2_O_2_ were incubated in 50-mM sodium acetate buffer pH 5.0 under standard, i.e., aerobic, conditions. At each time point, 100-µL samples were withdrawn, filtered and analyzed with HPAEC–PAD to quantify the release of oxidized products.

### Analysis of oxidized LPMO products with HPAEC–PAD

Filtrates of the reactions were analyzed by HPAEC–PAD on a Dionex ICS-5000 system (Sunnyvale, CA, USA) equipped with a CarboPac PA1 guard column (2 × 50 mm) and a CarboPac PA1 analytical column (2 × 250 mm). The flow was set at 0.25 mL/min, with an injection volume of 10 µL. For elution, two mobile phases were used: 0.1-M NaOH as eluent A, 1-M NaOAc in 0.1-M NaOH as eluent B. The following gradient was applied: linear increase from 0 to 10% B in 10 min, then to 30% B in the next 15 min, followed by an exponential gradient to 100% B in 5 min. The column was reconditioned before every injection by running 0% B for 9 min. Assignments of C1- and C4-oxidized peaks was based on comparison with products released from reference enzymes performing either C1- or C4-oxidation (*Nc*LPMO9F and *Nc*LPMO9C, respectively), which were produced as described elsewhere [45]. Gluconic acid and cellobionic acid were assigned and quantified using available standards (purchased from Megazyme and Synthose, respectively). To analyze the chromatograms, Chromeleon version 7.0 software (Thermo Fisher Scientific, Waltham, MA, USA) was used.

### Saccharification in bioreactors

Acid pretreated corn stover was obtained from SEKAB E-Technology, Örnsköldsvik, Sweden. The pretreatment was conducted in a pilot scale reactor, operated at steady-state conditions of 182 °C, 4.7 min residence time and an effective H_2_SO_4_ acid concentration of 0.35% (w/w) in the liquid, aiming at a pH of 2.5 [74]. The composition of this material is reported in Tables S4–S7 in Additional file 1. Saccharification was conducted in 1.5-L bioreactors (Infors HT Minifors) at 50 °C for 140 h, at pH 5.0 (controlled through automatic addition of 4 N NaOH) and using a mixing speed of 150 rpm. Industrial conditions were downscaled to a working volume of 1 L with 10% (w/w) dry matter, and saccharification was started by addition of a mixture of Celluclast^®^ (SigmaAldrich) (at 2.5 mg/gberk dry matter) and an in-house produced *A. niger* β-glucosidase (at 300 U/g dry matter), with or without LPMO (at 0.1 mg/g dry matter). Feedstock slurries were premixed under constant N_2_ for 16 h to ensure similar conditions in all reactors prior to enzyme loading. Reactions were carried out either aerobically or anaerobically through constant sparging of the headspace with air (labeled in figures as O_2_) or N_2_. To avoid microbial contamination, 0.2-µm filter disks were installed at the gas inlets and outlets. To confirm anaerobic conditions, an external oxygen sensor (Mettler-Toledo, Greifensee, Switzerland) was used to check the absence of dissolved oxygen in the liquid phase during saccharification. For the reactions with H_2_O_2_, hydrolysis was performed under N_2_, and H_2_O_2_ was added 3 times per day to a final concentration of 100–200 µM per addition. Samples were collected at different time points and immediately inactivated by addition of 4 N NaOH. Glucose released during saccharification was analyzed by high-performance liquid chromatography (HPLC) on a Dionex Ultimate 3000 (Dionex, Sunnyvale, USA). Products were separated using an Aminex^®^ HPX-87P 300 × 7.8-mm column, with ultrapure water as eluent at a flow of 0.6 mL/min and a temperature of 85 °C. Hydrolysis reactions were performed in duplicate.

### Thermal stability

The thermal stability of *Taus*LPMO9B was assessed using a thermal shift assay kit (Thermofisher, Massachusetts, USA) at pH ranging from 3.0 to 8.0 in different buffers (sodium citrate–phosphate for pH 3.0, sodium acetate for pH 5.0, BisTris/HCl for pH 7.0 and Tris/HCl for pH 8.0). Reactions containing 10-µM LPMO in each buffer and reference dye were prepared in a 96 well plate (Bio-rad). The plate was sealed with an optical adhesive film before incubation in a C1000 Dx Thermal Cycler with CFX96 Deep Well Dx Optical Reaction Module (Bio-Rad). The plate was heated from 25 to 95 °C with stepwise increments of 0.5 °C per min each followed by a 30-s hold, while fluorescence, with an excitation/emission wavelength at 488/602 nm, was monitored. All reactions were carried out in triplicate.

### Peptide map LC–MS/MS

50 µl sample was added to 450-µl 100-mM ammonium bicarbonate (Sigma Aldrich) and 5-µl 500-mM dithiothreitol (DTT, Sigma Aldrich) in 100-mM ammonium bicarbonate. The cysteines were reduced by 30-min incubation at 55 °C. 5-µl 550-mM freshly prepared iodoacetamide (IAA, Sigma Aldrich) in 100-mM ammonium bicarbonate was added, cysteines were alkylated by 30-min incubation at 20 °C. The reaction was quenched by addition of 1-µl 500-mM DTT in 100-mM ammonium bicarbonate. 20-µl 0.25-µg/µl trypsin gold (Promega, Madison WI, USA) in 5-mM HCl was added, and the sample was digested by 4-h incubation at 37 °C. Another 4-µl 0.25-µg/µl trypsin gold was added followed by 1-h incubation at 37 °C to ensure completion of the digestion. The sample was split into three 100-µl fractions: (1) one part was acidified with 1-µl formic acid and analyzed directly; (2) one part was digested with 1-µl 1-U/µl PNGase F (Promega, Madison WI, USA) for 1 h at 37 °C, then acidified with 1-µl formic acid and analyzed; and (3) one part was acidified with 10-µl 5% formic acid to pH ~ 5, then digested with 10-µl 5-U/mL Endo Hf (Roche Applied Science, Penzberg, Germany) for 1 h at 37 °C, then acidified further with 1-µl formic acid and analyzed.

Samples were analyzed by liquid chromatography–tandem mass spectrometry (LC–MS/MS). The system consisted of a Vanquish UHPLC coupled to a Q Exactive Plus Orbitrap MS (Thermo Scientific, San Jose, CA, USA) using a 2.1 × 100 mm 1.7-µm CSH column (Waters Corporation, Milford, MA, USA); the column oven was set to 50 °C, the injection volume was 10 µl, and the flow rate was set to 0.4 mL/min. Peptides were separated using reverse-phase chromatography using a gradient of water with 0.1% formic acid (solvent A) and 90% acetonitrile with 0.1% formic acid (solvent B) from 6.3% B to 55% B in 10 min, total runtime of 15 min. Data-dependent acquisition (DDA) was performed with a resolution setting at 35,000 within the *m/z* range 400–1,600 and a maximum injection time of 75 ms, followed by high-energy collision-induced dissociation-activated (HCD) MS/MS on the top 5 most abundant precursors using a resolution setting of 17,500 and a *m/z* range 50–2,000 with a maximum injection time of 50 ms. The minimum intensity threshold for MS/MS was 1,000 counts, and peptide species with 1 and > 8 charges were excluded. MS/MS spectra were analyzed with the MS Amanda search engine in Proteome Discoverer, version 2.3, against the *A. niger* database (in-house) and the complete and mature *Taus*LPMO9B sequence, lacking the signal peptide. Protein N-terminal methylation, deamidation (N/Q), oxidation (M) were set as variable modification, and carbamidomethylation (C) was set as fixed modification. Precursor mass accuracy was set to 10 ppm and fragment mass accuracy to 0.02 Da. Trypsin was used as cleavage preference allowing up to 3 missed cleavages. Fixed Value PSM Validator was used, and only high confident peptide matches were considered for the peptide map.

### Intact protein LC–MS

The sample was diluted to 50 µg/mL in water containing 2% (w/v) formic acid, prior to LC–MS analysis. LC–MS was performed using an ACQUITY I-class UPLC coupled to a Synapt G2S quadrupole-time-of-flight (Q-TOF) mass spectrometer (Waters Corporation, USA). 5 µl was injected into a C4 analytical column (Acquity UPLC Protein BEH300 1.7 µm 300 Å pore size 2.1 × 50 mm, Waters Corporation, USA). The column temperature was set to 75 °C. As mobile phase, a gradient of premixed 0.1% formic acid in water (A), and premixed 0.1% formic acid in acetonitrile (B) was used at 400 µl/min. The first 2 min was kept isocratic at 97% A, followed by a decrease in the percentage of mobile phase A to 83% in 0.2 min. From 2.2 to 8.0 min, the percentage A was further decreased to 56%, after which the column was kept at 56% A for 2 min, followed by an increase to 97% A in 10 min. Mass calibration was performed over a mass range (*m/z*) of 500–3500 using sodium-trifluoroacetate clusters. Mass correction on the flight was performed using leucine enkephalin (Sigma) as lockmass. The mass spectra were recorded in continuum resolution mode. MS settings used are hereby listed: Capillary 1 kV, Sampling Cone 40, Source Offset 80, Source Temperature 120 °C, Desolvation Temperature 400 °C, Cone Gas 50 L/h, Desolvation Gas 700 L/h, and Nebuliser Gas 6 Bar. The molecular mass of the protein was calculated by maximum entropy deconvolution using MaxEnt2, using the following parameters: Ranges 20,000:45,000, Resolution 0.9 Da/channel (could be varied), Damage model Uniform Gaussian 0.75 Da, and iteration to convergence.

## Supplementary information

**Additional file 1.** Tables and figures.

## Data Availability

The dataset(s) supporting the conclusions of this article is(are) included within the article (and its additional file(s)).

## Abbreviations

AA: Auxiliary activity
CAZymes: Carbohydrate Active enZymes
CSL: Corn steep liquor
DM: Dry matter
DP: Degree of polymerization
GH: Glycoside hydrolase
HPLC: High-performance liquid chromatography
HPAEC–PAD: High-performance anion exchange chromatography with pulsed amperometric detection
LPMO: Lytic polysaccharide monooxygenase
PASC: Phosphoric acid swollen cellulose
PCS: Pretreated corn stover
PTM: Post-translational modification
