# Supplementary material for: Characterization of an AA9 LPMO from Thielavia australiensis, TausLPMO9B, under industrially relevant lignocellulose saccharification conditions
Source: Biotechnol Biofuels. 2020 Nov 30;13:195. doi: 10.1186/s13068-020-01836-3 (PMC7706046; doi:10.1186/s13068-020-01836-3)
Supplement: Supplementary file 1 — Additional file 1. Tables and figures. [file 13068_2020_1836_MOESM1_ESM.docx]

Characterization of an AA9 LPMO from *Thielavia australiensis,* *Taus*LPMO9B, under industrially relevant lignocellulose saccharification conditions

F. Calderaro^1,2*^, M. Keser^1^, M. Akeroyd^1^, L.E. Bevers^1^, V.G.H. Eijsink^3^, A. Várnai^3^, M.A. van den Berg^1^

**Supporting information**

# Additional tables

**Table S1** The amino acid sequence of TausLPMO9B. The sequence indicated includes the signal peptide (highlighted in grey), the AA9 domain (highlighted in yellow) and the CBM1 domain (highlighted in green). The N-terminal end of the linker containing three Cys residues is underlined.

| **Enzyme** | **Sequence** |
| --- | --- |
| *Taus*LPMO9B | MKSFTVAALAALWAQNAAAHATFQDLWIDGVDYGSQCARLPASNNPVTDVSSNDIRCNIIGTHPGAKCPVKAGSTVTVEMHQQNGDRSCANEAIGGAHWGPVMVYMSKVSDAATADGSSGWFKVFQDTWAKNPSGYSGDDDYWGTKDLNQCCGKMNVKIPSDLPSGDYLLRAEVIALHVAGSTGGAQFYMTCYQLTVTGGGSASPSTVSFPGAYKASDPGILVNIHAPMSTYVAPGPAVYSGGSTKSAGSSCSGCEATCTPGSGPSPTLSQPPPSTSTGSAPGGGGTSGCTVQKYGQCGGTGYTGCTTCASGSTCSAVSPPYYYQCI |

**Table S2** Highest hits in protein-Blast (blastp) search against the non-redundant NCBI database using the full sequence of TausLPMO9B as a query sequence.

| **Accession** | **Organism** | **Protein** | **UniProt** | **Sequence identity (%)** | **Domains** |
| --- | --- | --- | --- | --- | --- |
| XP_003663414.1 | *Myceliophthora thermophila* | MYCTH_80312 (*Mt*LPMO9B) | G2QCJ3 | 82.0 | AA9, CBM1 |
| KXX81265.1 | *Madurella mycetomatis* | MMYC01_203185 | A0A175WCB1 | 79.2 | AA9, CBM1 |
| XP_003655380.1 | *Thielavia terrestris* | THITE_2119040  (GH61B) | G2RB73 | 82.8 | AA9 |
| SPQ22679.1 | *Thielavia terrestris* | TT172_LOCUS5098 | A0A3S4B613 | 83.1 | AA9 |
| OIW35682.1 | *Coniochaeta ligniaria* | CONLIGDRAFT_609141 (CBM1 domain-containing protein) | A0A1J7K608 | 75.7 | AA9, CBM1 |
| [KAB5530284.1](https://www.ncbi.nlm.nih.gov/protein/KAB5530284.1?report=genbank&log$=prottop&blast_rank=6&RID=8FC8Y9S4016) | *Coniochaeta* sp. 2T2.1 | GE09DRAFT_386228 (GH61 domain-containing protein) | A0A5N5KIW9 | 76.8 | AA9, CBM1 |
| KAB5581128.1 | *Coniochaeta* sp. 2T2.1 | GE09DRAFT_463781 (GH61 domain-containing protein) | A0A5N5PPM4 | 77.1 | AA9, CBM1 |
| XP_001229931.1 | *Chaetomium globosum* | CHGG_03415 (CBM1 domain-containing protein) | Q2H8N9 | 76.8 | AA9, CBM1 |
| RKU46439.1 | *Coniochaeta pulveracea* | DL546_006773 (CBM1 domain-containing protein) | [A0A420YES8](https://www.uniprot.org/uniprot/A0A420YES8) | 75.4 | AA9, CBM1 |

**Table S3** The complete list of identified peptides in the peptide map of TausLPMO9B after tryptic digestion and LC-MS analysis. Only high confident peptide matches are shown; these peptides cover >70% of the sequence of TausLPMO9B. The table shows the retention time (RT), m/z values, the annotated sequence, modifications detected, the number of peptide-spectrum matches (PSMs), position of the peptide in the protein sequence, the missed cleavages and the theoretical mass. The two C-terminal peptides expected after trypsin digestion were not identified, most likely because they are large and/or carry O-glycosylations.

| **RT (min)** | ***m/z*** | **Annotated Sequence** | **Modifications** | **# PSMs** | **Position** | **Missed Cleavages** | **Theo. M+H^+^ [Da]** |
| --- | --- | --- | --- | --- | --- | --- | --- |
| 3.67 | 497.752 | VFQDTWAK |  | 2 | [105-112] | 0 | 994.499 |
| 4.31 | 899.946 | LPASNNPVTDVSSNDIR |  | 5 | [21-37] | 0 | 1798.893 |
| 4.42 | 749.841 | VSDAATADGSSGWFK |  | 5 | [90-104] | 0 | 1498.681 |
| 6.23 | 1177.033 | HATFQDLWIDGVDYGSQCAR | 1xCarbamidomethyl [C18]; 1xMethyl [N-term] | 8 | [1-20] | 0 | 2353.067 |
| 5.73 | 1133.011 | SCANEAIGGAHWGPVMVYMSK | 1xCarbamidomethyl [C2] | 4 | [69-89] | 0 | 2265.025 |
| 3.83 | 831.335 | NPSGYSGDDDYWGTK |  | 4 | [113-127] | 0 | 1661.671 |
| 6.31 | 1019.847 | ASDPGILVNIHAPMSTYVAPGPAVYSGGSTK |  | 4 | [197-227] | 0 | 3057.535 |
| 2.15 | 577.597 | AGSTVTVEMHQQNGDR | 1xDeamidated [N/Q] | 32 | [53-68] | 0 | 1730.776 |
| 1.06 | 584.298 | CNIIGTHPGAK | 1xCarbamidomethyl [C1] | 2 | [38-48] | 0 | 1167.594 |
| 5.79 | 723.384 | IPSDLPSGDYLLR |  | 2 | [140-152] | 0 | 1445.763 |
| 1.44 | 577.267 | AGSTVTVEMHQQNGDR |  | 11 | [53-68] | 0 | 1729.792 |
| 6.2 | 780.352 | HATFQDLWIDGVDYGSQCAR | 1xCarbamidomethyl [C18] | 3 | [1-20] | 0 | 2339.051 |
| 5.18 | 761.01 | SCANEAIGGAHWGPVMVYMSK | 1xCarbamidomethyl [C2]; 1xOxidation [M] | 6 | [69-89] | 0 | 2281.02 |
| 4.47 | 900.439 | LPASNNPVTDVSSNDIR | 1xDeamidated [N] | 6 | [21-37] | 0 | 1799.877 |
| 7.24 | 936.927 | SCANEAIGGAHWGPVMVYMSKVSDAATADGSSGWFK | 1xCarbamidomethyl [C2] | 3 | [69-104] | 1 | 3744.688 |
| 5.95 | 756.006 | SCANEAIGGAHWGPVMVYMSK | 1xCarbamidomethyl [C2]; 1xDeamidated [N4] | 1 | [69-89] | 0 | 2266.009 |
| 5.78 | 1025.177 | ASDPGILVNIHAPMSTYVAPGPAVYSGGSTK | 1xOxidation [M14] | 1 | [197-227] | 0 | 3073.53 |

**Table S3** (continued)

| **RT (min)** | ***m/z*** | **Annotated Sequence** | **Modifications** | **# PSMs** | **Position** | **Missed Cleavages** | **Theo. M+H^+^ [Da]** |
| --- | --- | --- | --- | --- | --- | --- | --- |
| 8.28 | 944.841 | SCANEAIGGAHWGPVMVYMSKVSDAATADGSSGWFKVFQDTWAK | 1xCarbamidomethyl [C2] | 1 | [69-112] | 2 | 4720.169 |
| 7.25 | 825.392 | VSDAATADGSSGWFKVFQDTWAK |  | 1 | [90-112] | 1 | 2474.162 |
| 2.21 | 554.263 | CPVKAGSTVTVEMHQQNGDR | 1xCarbamidomethyl [C1] | 2 | [49-68] | 1 | 2214.039 |
| 7.1 | 1110.538 | AEVIALHVAGSTGGAQFYMTCYQLTVTGGGSASPSTVSFPGAYK | 1xCarbamidomethyl [C21] | 2 | [153-196] | 0 | 4439.132 |
| 7.2 | 780.682 | HATFQDLWIDGVDYGSQCAR | 1xCarbamidomethyl [C18]; 1xDeamidated [Q5] | 2 | [1-20] | 0 | 2340.035 |
| 3.98 | 879.69 | NPSGYSGDDDYWGTKDLNQCCGK | 2xCarbamidomethyl [C20; C21] | 1 | [113-135] | 1 | 2637.062 |
| 2.5 | 554.512 | CPVKAGSTVTVEMHQQNGDR | 1xCarbamidomethyl [C1]; 1xDeamidated [N17] | 1 | [49-68] | 1 | 2215.023 |

**Table S4** Weight fractions of acid pretreated corn stover (g/kg wet material)

| **Total solids** | **Insoluble solids** | **Ash content in  Total solids** | **Ash content in  Insoluble solids** |
| --- | --- | --- | --- |
| 215.0 ± 7.7 | 136.5 ± 1.1 | 22.9 ± 0.8 | 12.8 ± 0.1 |

**Table S5** Total sugar composition of acid pretreated corn stover (g/kg wet material)

| **Total sugars**  **(anhydro form)** | **Insoluble sugars**  **(anhydro form)** | **Soluble oligosaccharides**  **(anhydro form)** | **Soluble monosaccharides**  **(monomers)** |
| --- | --- | --- | --- |
| 120.2 ± 0.3 | 76.0 ± 0.7 | 19.9 ± 1.3 | 27.2 ± 2.1 |

**Table S6** Polysaccharide composition of acid pretreated corn stover (g/kg wet material)

|  | **Galactan** | **Xylan** | **Mannan** | **Glucan** | **Arabinan** |
| --- | --- | --- | --- | --- | --- |
| **Sugar (g/kg)** | 2.5 ± 0.3 | 33.6 ± 0.3 | 1.0 ± 0.2 | 79.0 ± 0.4 | 4.2 ± 0.1 |
| **Insoluble sugar (g/kg)** | 0.2 ± 0.1 | 6.0 ± 0.3 | 0.3 ± 0.1 | 68.9 ± 0.5 | 0.5 ± 0.1 |
| **Soluble oligomers (g/kg)** | 0.9 ± 0.1 | 11.9 ± 1.0 | 0 | 3.9 ± 0.2 | 3.2 ± 0.1 |

**Table S7** Soluble monosaccharide composition of acid pretreated corn stover (g/kg wet material)

| **Galactose** | **Xylose** | **Mannose** | **Glucose** | **Arabinose** |
| --- | --- | --- | --- | --- |
| 1.7 ± 0.2 | 16.9 ± 1.5 | 0.8 ± 0.1 | 4.0 ± 0.3 | 3.8 ± 0.2 |

# Additional figures


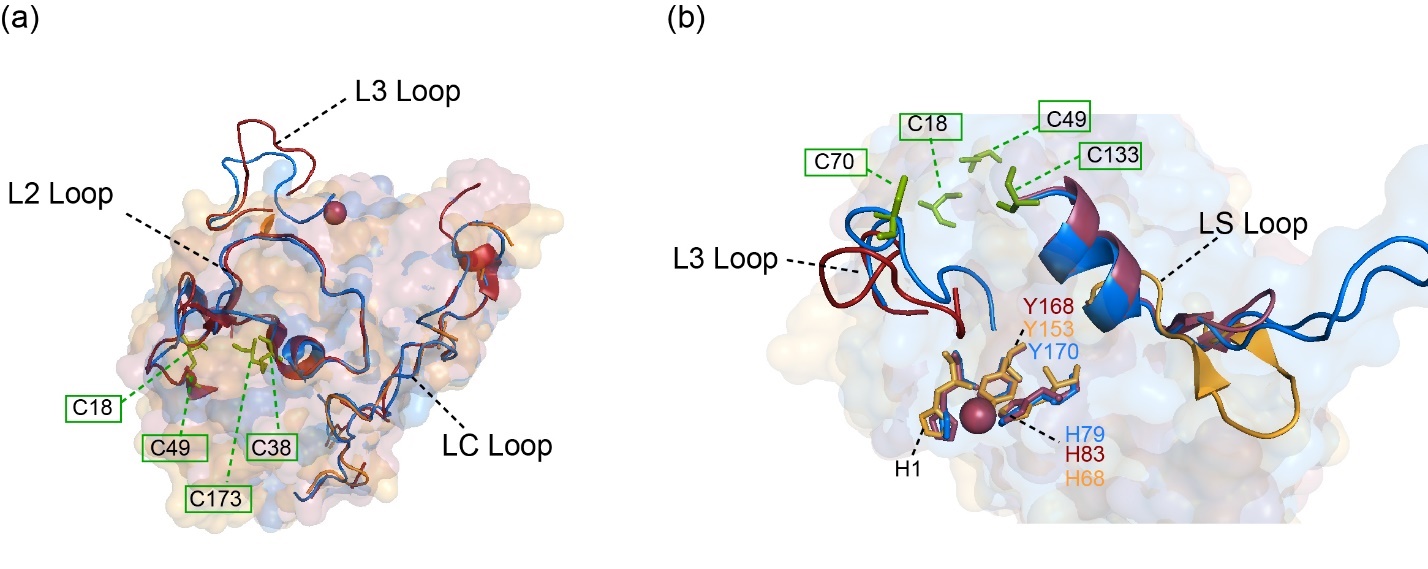


**Figure S1** 3D structural model of TausLPMO9B built using the structure of NcLPMO9C (PDB, 4D7U) as a template. **a)** Structural superposition of the three-dimensional model of TausLPMO9B (blue) with the crystal structures of the C4-oxidizing NcLPMO9C (red; (1); PDB, 4D7U) and C1-oxidizing TtLPMO9E (orange; (2); PDB, 3EJA). The copper ion is shown as a purple sphere, and surface loops involved in shaping the substrate-binding surface are shown as cartoons. The cysteines involved in the formation of the two predicted disulfide bridges are indicated in green squares. Note that TtLPMO9E lacks the L3 loop. **b)** Close-up view of the catalytic center and potential disulfide bridges, with the same color coding as in panel **a**. The side chains shown are predicted to be involved in binding the copper and shaping the copper site. Note that the predictability of model is limited due to the low (<50%) sequence identity of TausLPMO9B and the model LPMO NcLPMO9C.

In agreement with the structural model for MtLPMO9B (UniProt ID, G2QCJ3) based on the NcLPMO9D structure (PDB ID, 5TKF; with 45% sequence identity) by Laurent et al. (3), our homology model predicts the formation of disulfide bonds between the Cys18–Cys49 pair (within the L2 loop) and the Cys38–Cys173 pair (the only two Cys residues that are highly conserved in all AA9 LPMOs). In addition, the model by Laurent et al. indicates a potential disulfide bridge between Cys70–Cys133. Cys70 (position 74 in Figure 1) is located in the extended L3 loop (referred to as Seg2 by Laurent et al.); Cys133 (and the adjacent Cys132; positions 129 and 128 in Figure 1) is located at one of the ends of the LS loop (referred to as Seg3 by Laurent et al.). Laurent et al. hypothesize that such a disulfide bridge would mimic a shorter L3 loop by pulling the L3 loop away from the substrate-binding surface and thus affect regioselectivity (3).


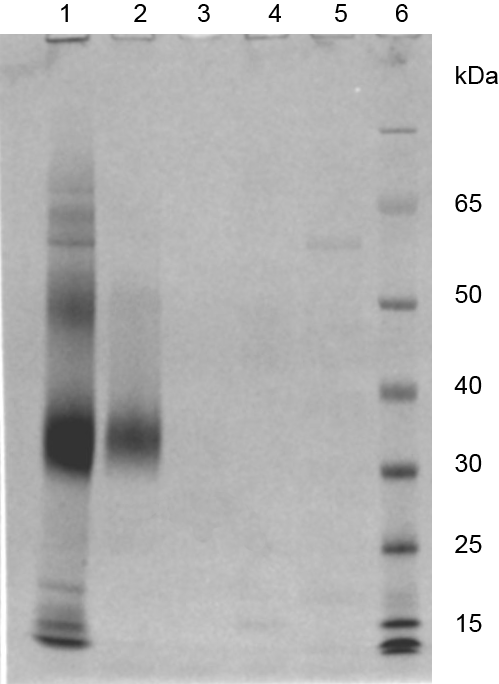


**Figure S2** SDS PAGE analysis of TausLPMO9B. Lane 1, fermentation sample before purification; Lane 2, fraction containing purified TausLPMO9B (theoretical molecular weight of 31.4 kDa); Lanes 3-5, additional fractions collected during purification; Lane 6, protein ladder.


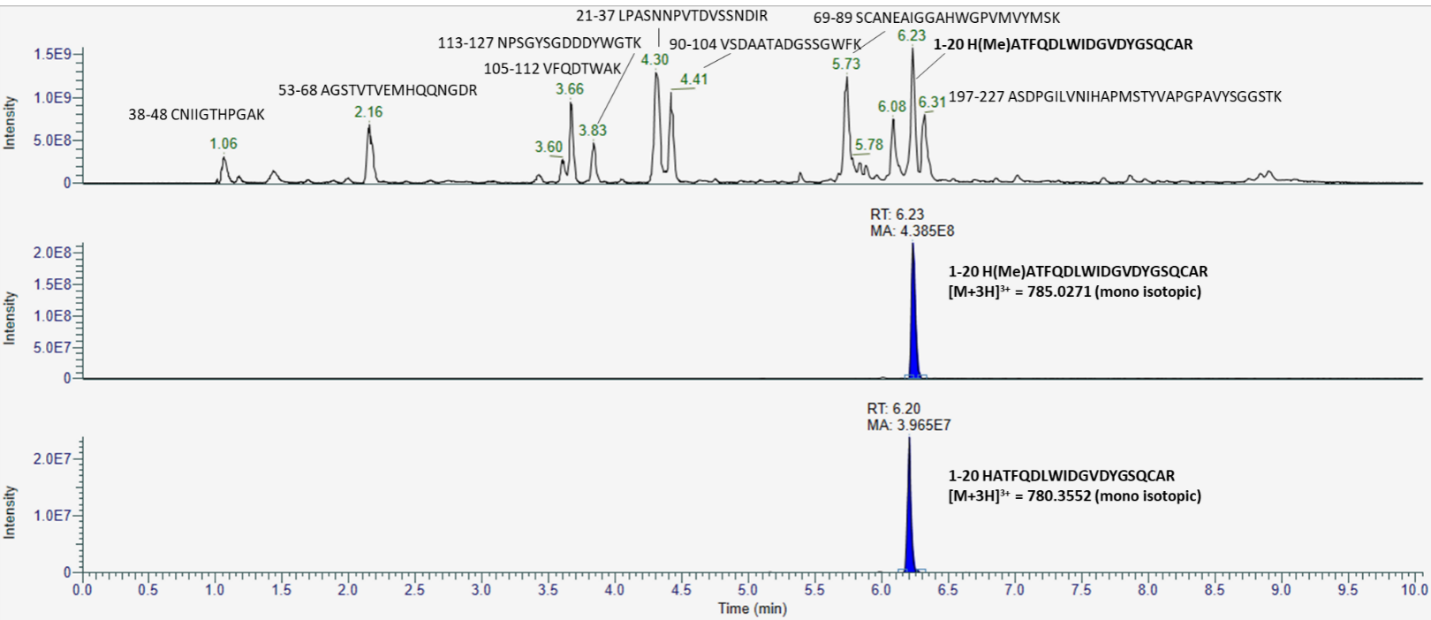


**Figure S3** LC-MS chromatogram of the peptide map of TausLPMO9B after digestion with trypsin. The figure shows the LC-MS chromatogram of the digested TausLPMO9B in the top panel. The main peaks and identified peptides in these peaks are indicated. The extracted ion chromatograms of the methylated N-terminal peptide (middle panel) and the non-methylated N-terminal peptide (bottom panel) are from the triply charged mono isotopic ions with 5 ppm accuracy. The MS/MS fragmentation spectra are shown in Fig. S4. The ratio of methylated versus non-methylated N-terminus is represented by the areas indicated in the extracted ion chromatograms and equals to 91.7:8.3.


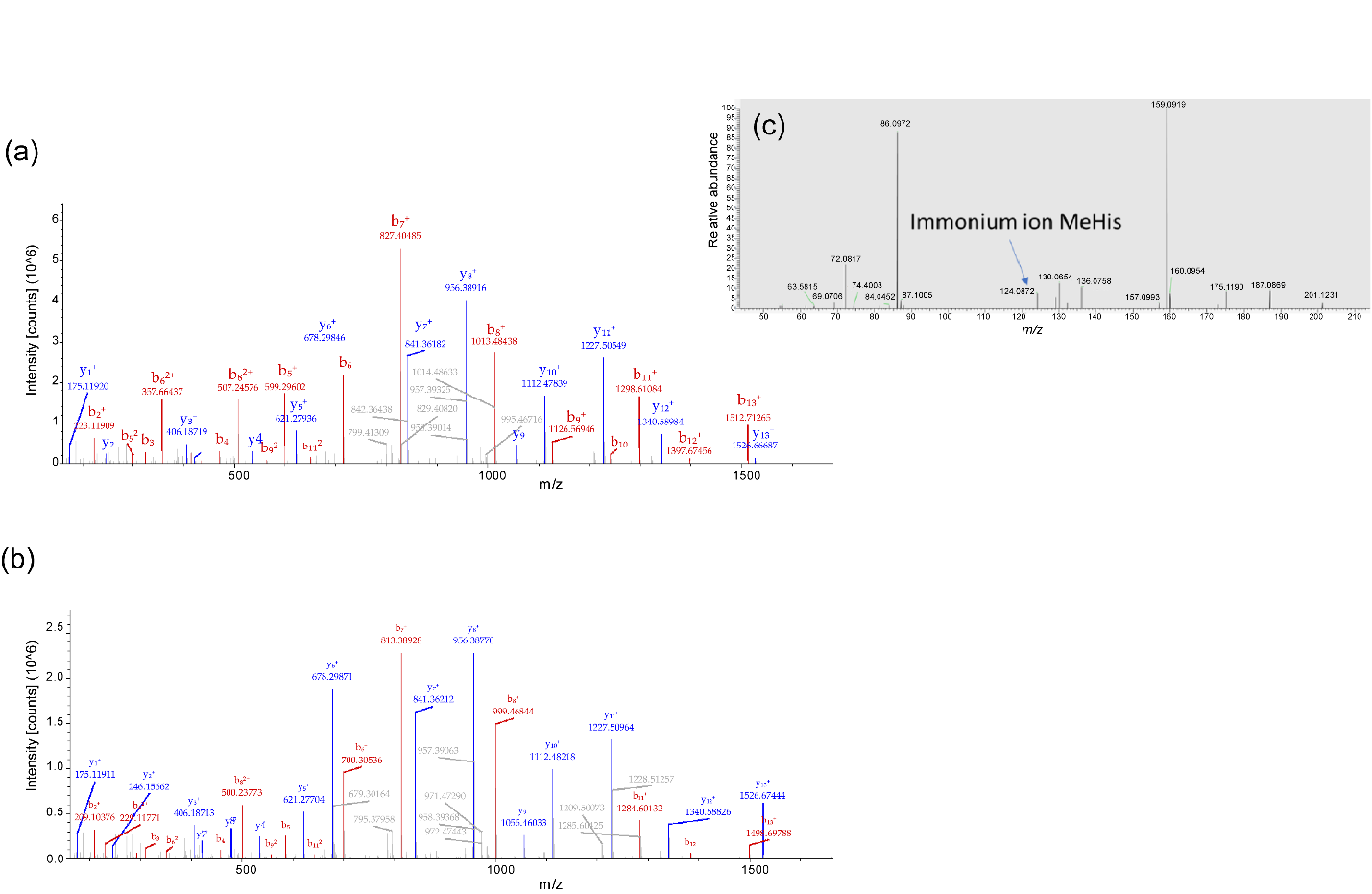


**Figure S4** The fragmentation spectra of the N-terminal peptide. **a)** MS/MS spectrum of the methylated N-terminal peptide **b)** MS/MS spectrum of the non-methylated peptide. The matched b ions are indicated in red, and the matched y ions are indicated in blue. **c)** zoom-in on the low m/z region of the methylated N-terminal peptide, the immonium ion of the methylated histidine is observed.


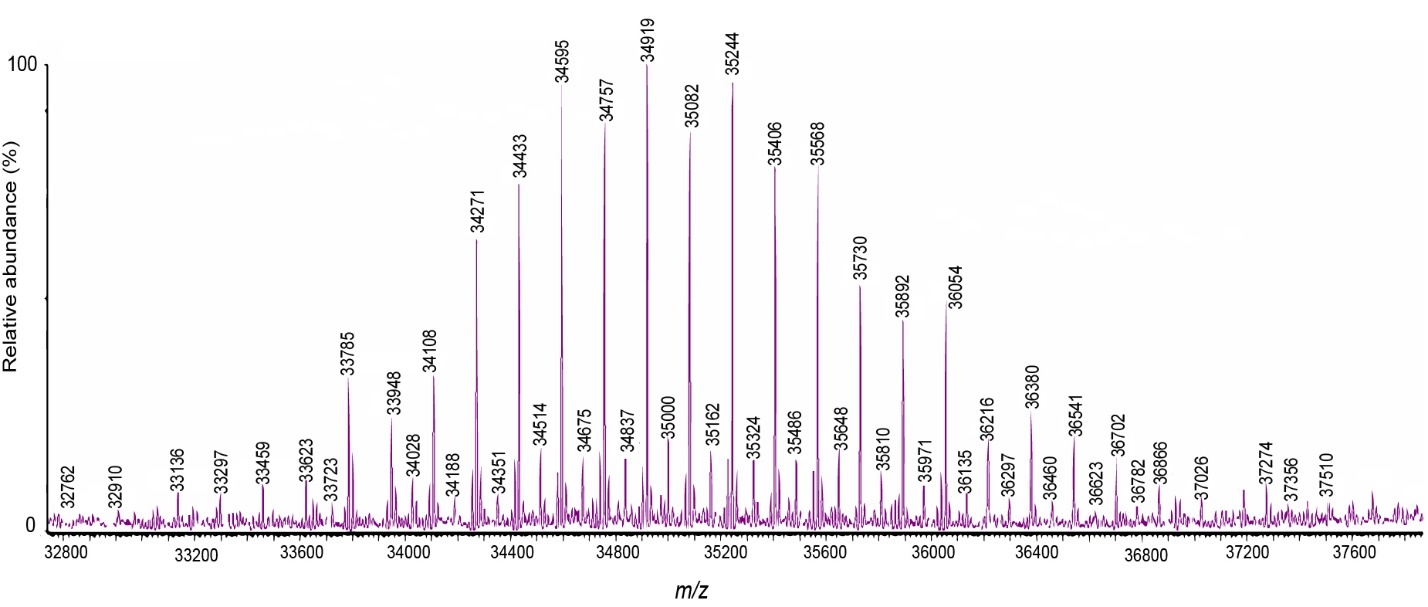


**Figure S5** Mass spectrum of intact *Taus*LPMO9B. The figure shows the deconvoluted mass spectrum of the main chromatographic peak observed for the *Taus*LPMO9B. The theoretical *m*/*z* value for the non-glycosylated, methylated protein is 31,355. The deconvoluted spectrum shows the main proteoforms of *Taus*LPMO9B ranging from *m/z* 33,136 to *m/z* 37,677 with increments of 162. These increments correspond to the residual mass of a hexose and indicate heavy and heterogeneous glycosylation of *Taus*LPMO9B.


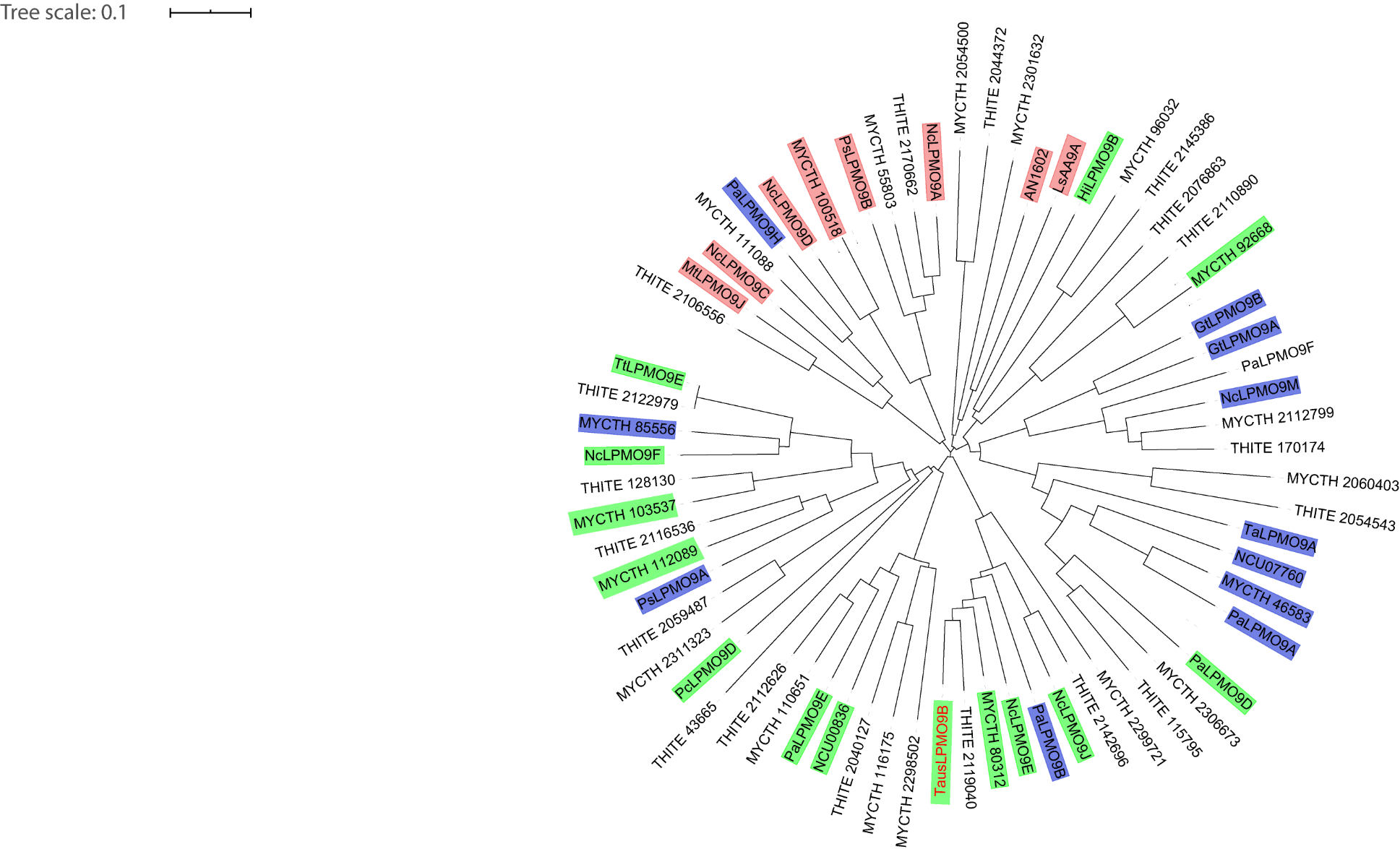


**Figure S6** Phylogenetic analysis of the AA9 domains of different LPMOs. The tree includes sequences from both characterized (colored background) and uncharacterized LPMOs (white background), and includes all LPMO9s from the thermophilic fungi Myceliophthora thermophila and Thielavia terrestris. C1-oxidizing LPMOs are indicated with green, C4-oxidizing LPMOs with red, and C1/C4-oxidizing LPMOs with blue background. TausLPMO9B is indicated with a green background and red label. The tree was built based on a multiple sequence alignment performed with Clustal Omega and visualized with the iTOL platform (4).

**
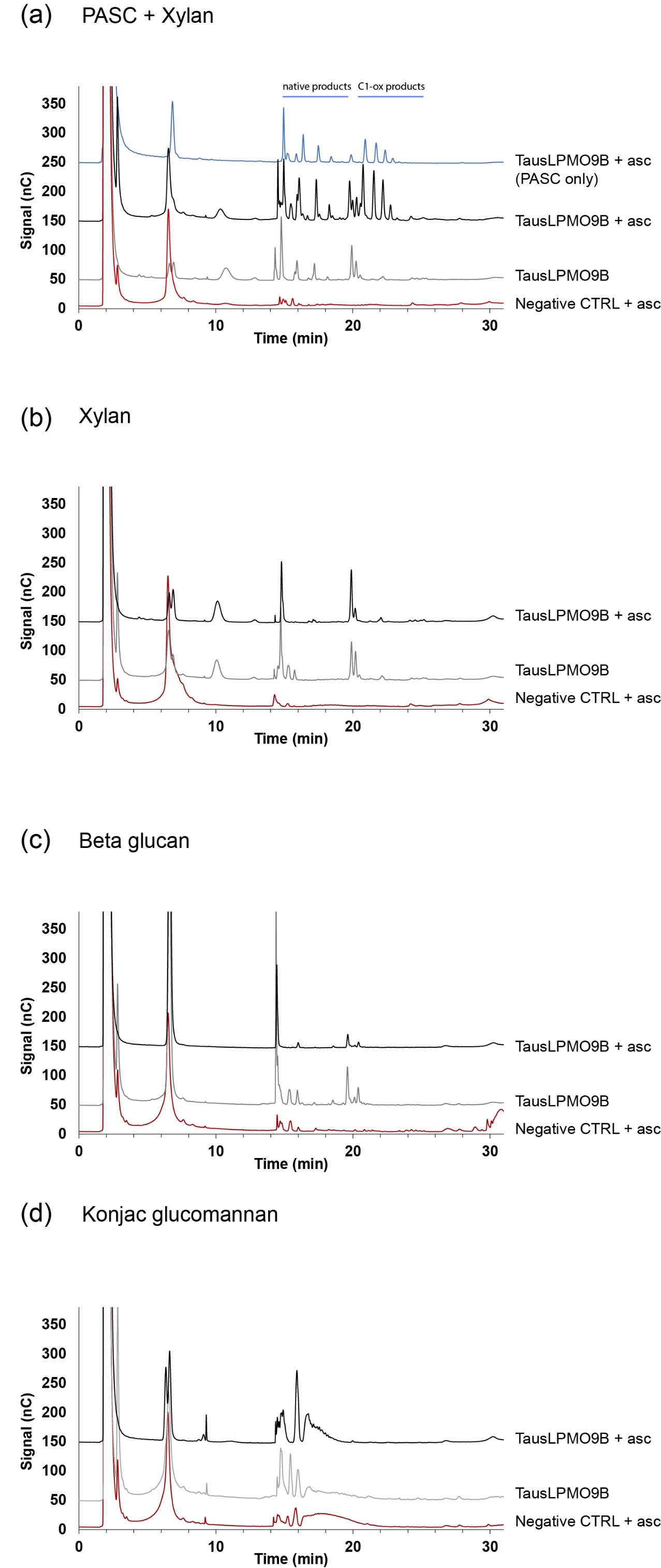
**
**
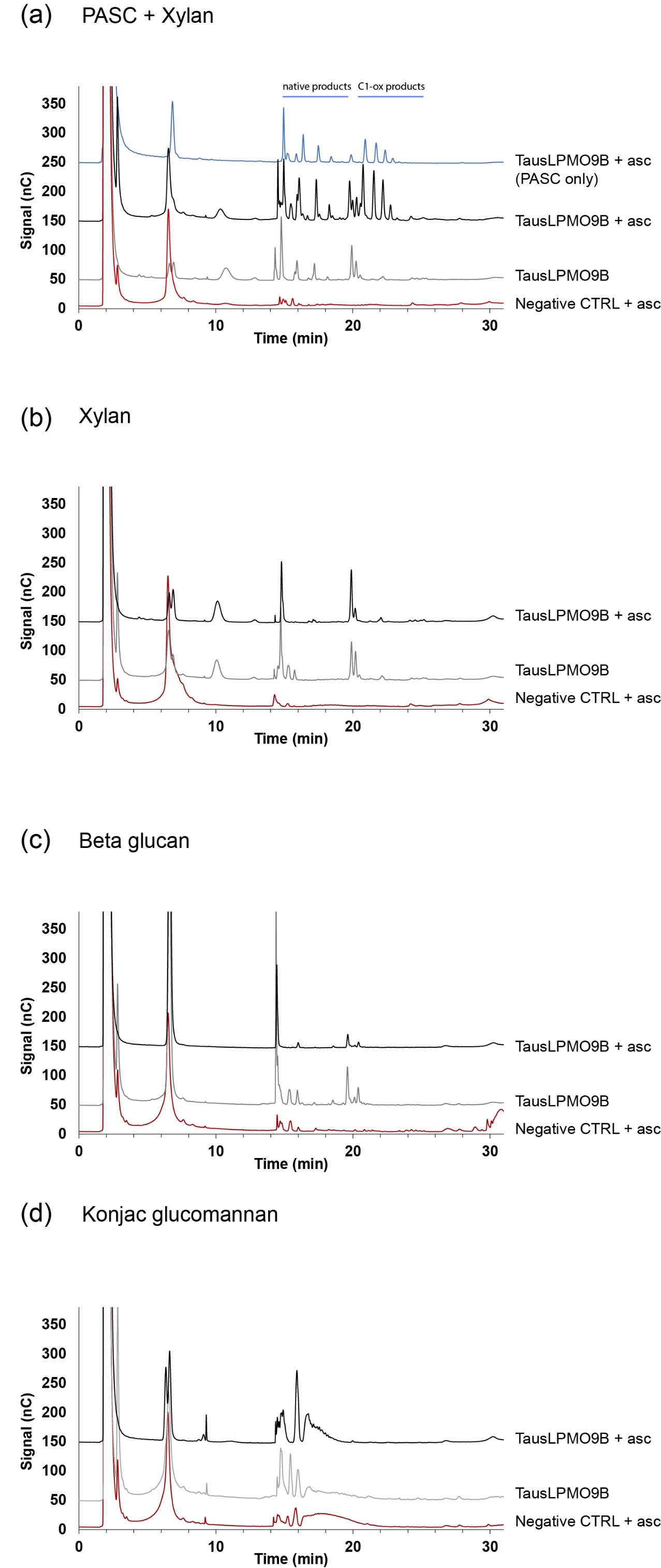
**

**
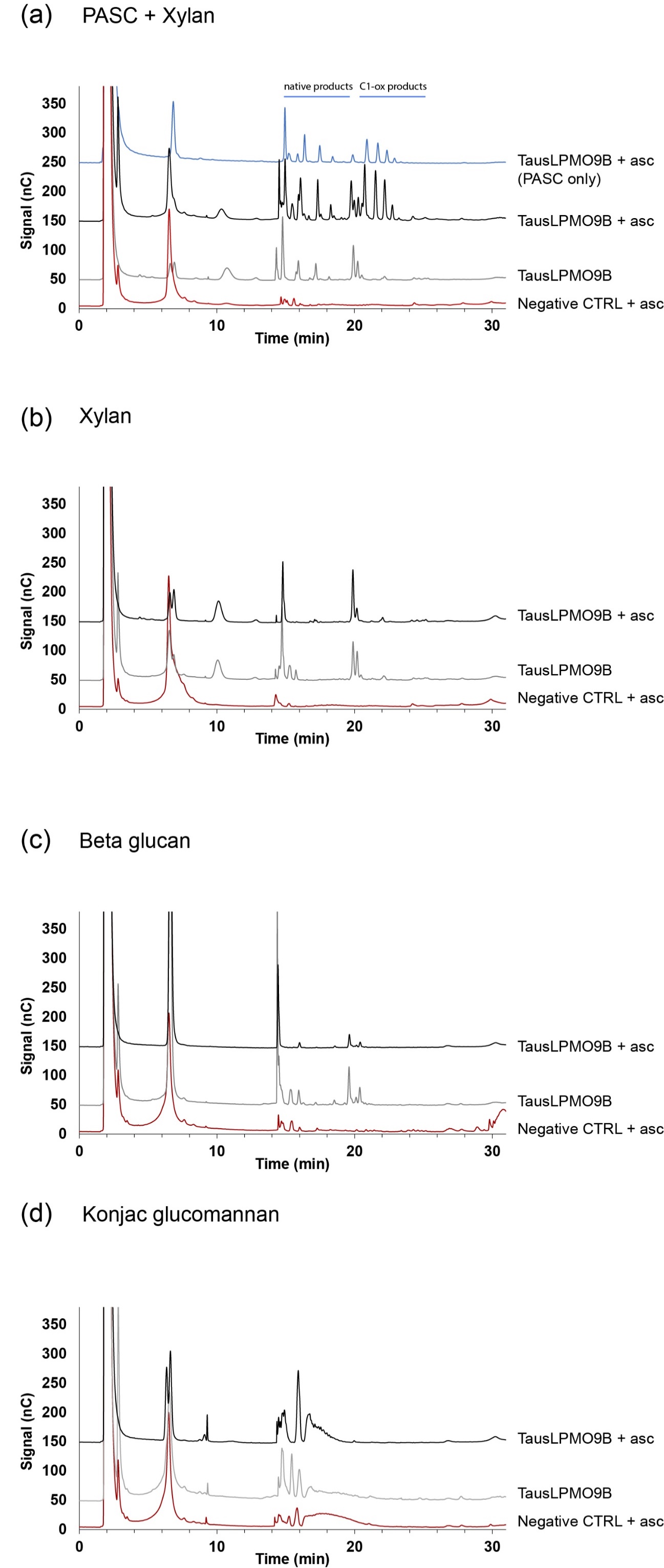


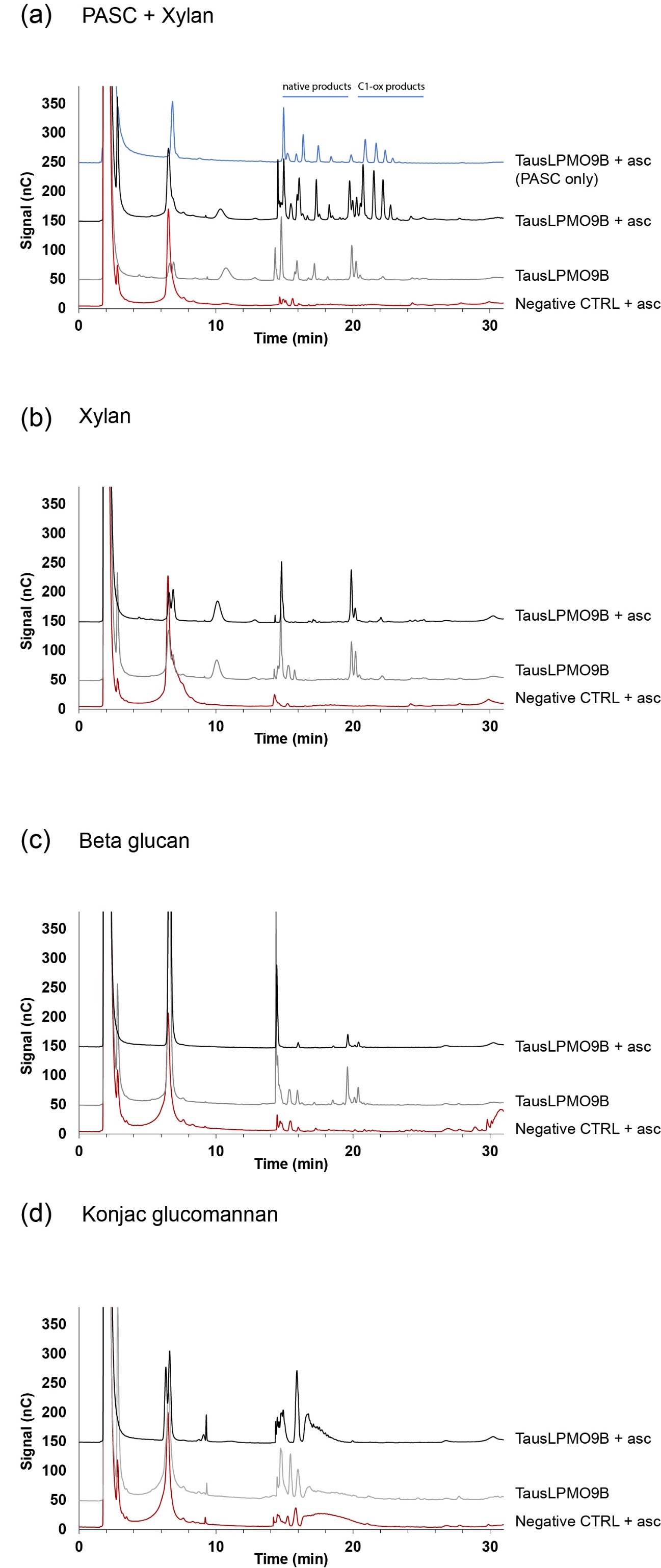
**

**Figure S7** (continued)

**
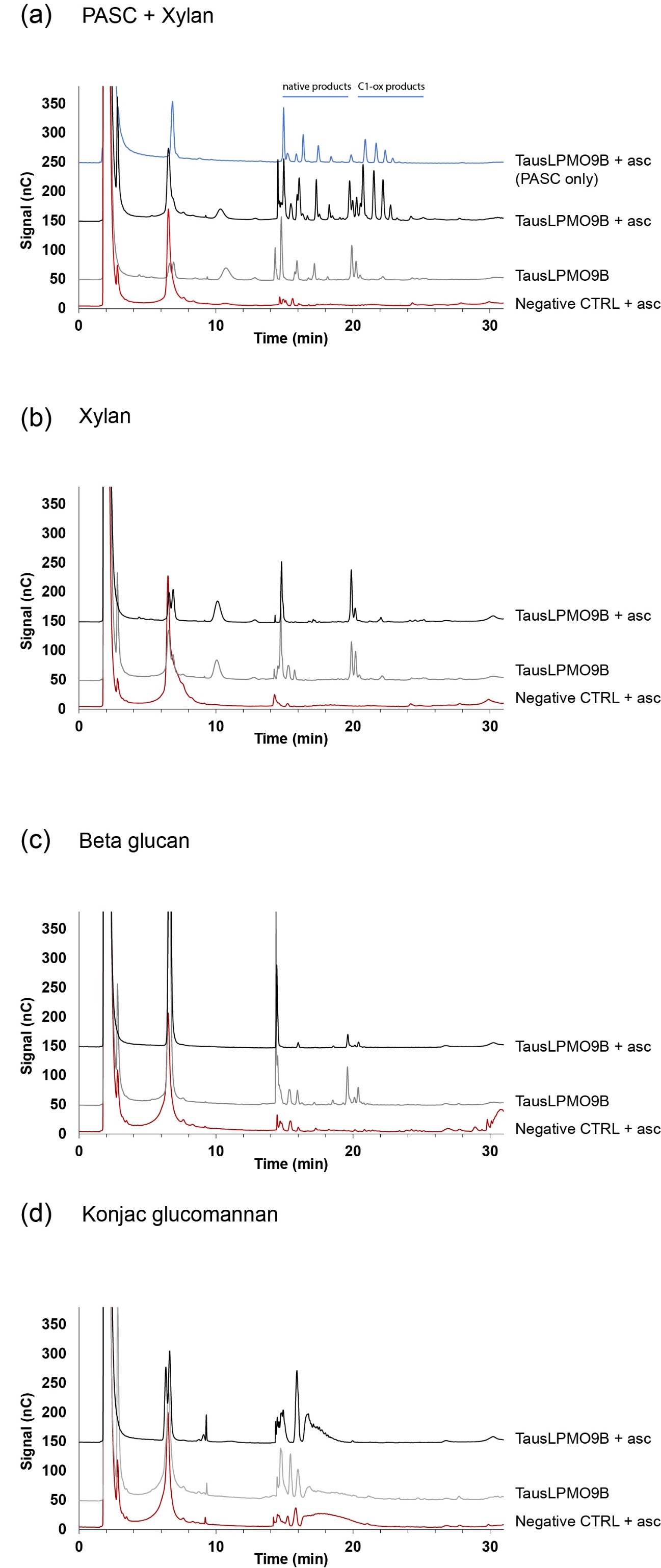


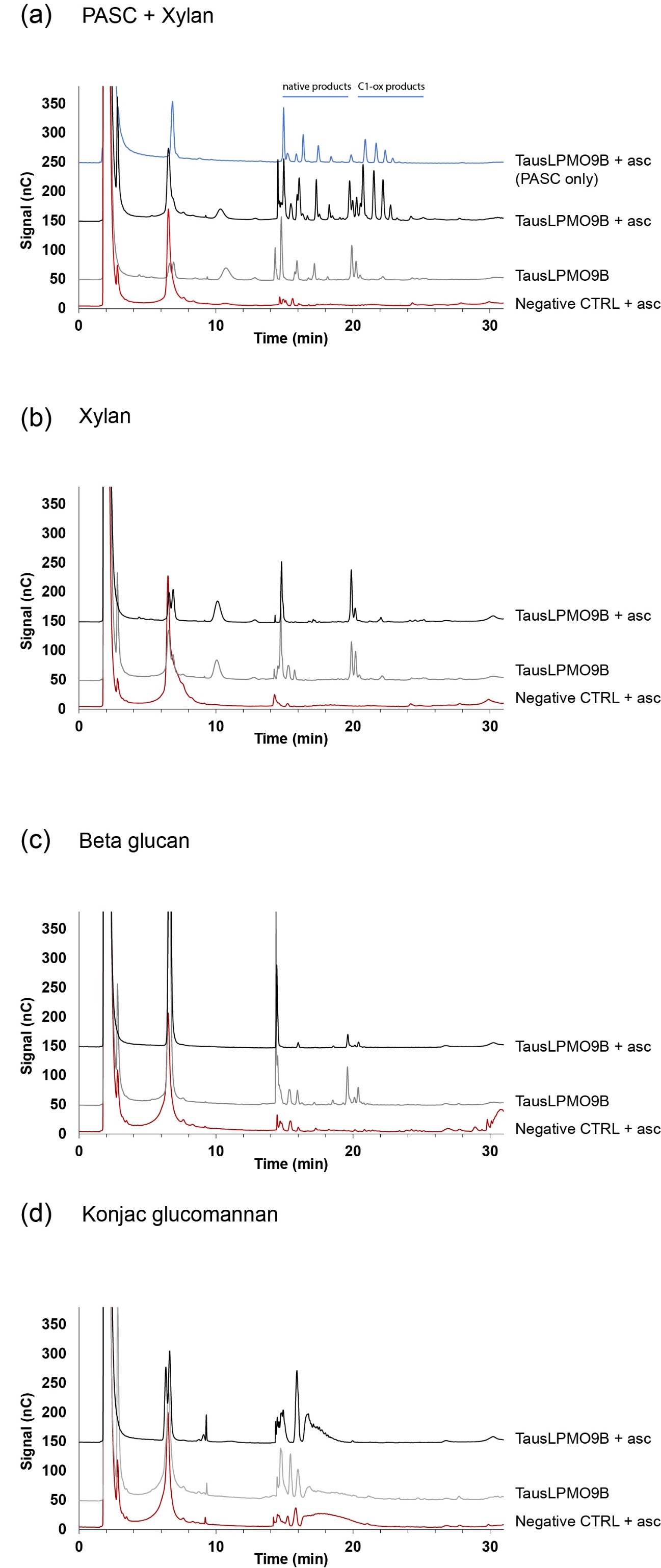
**

**
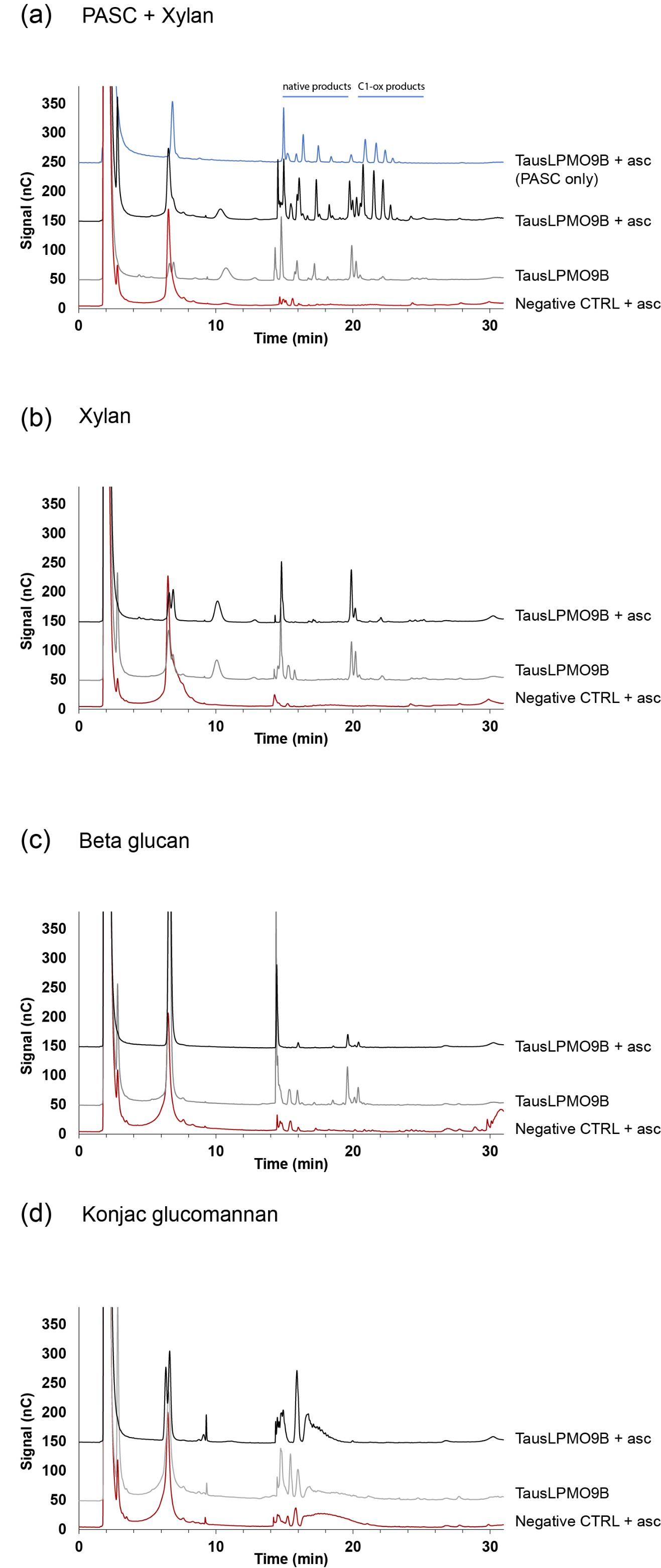


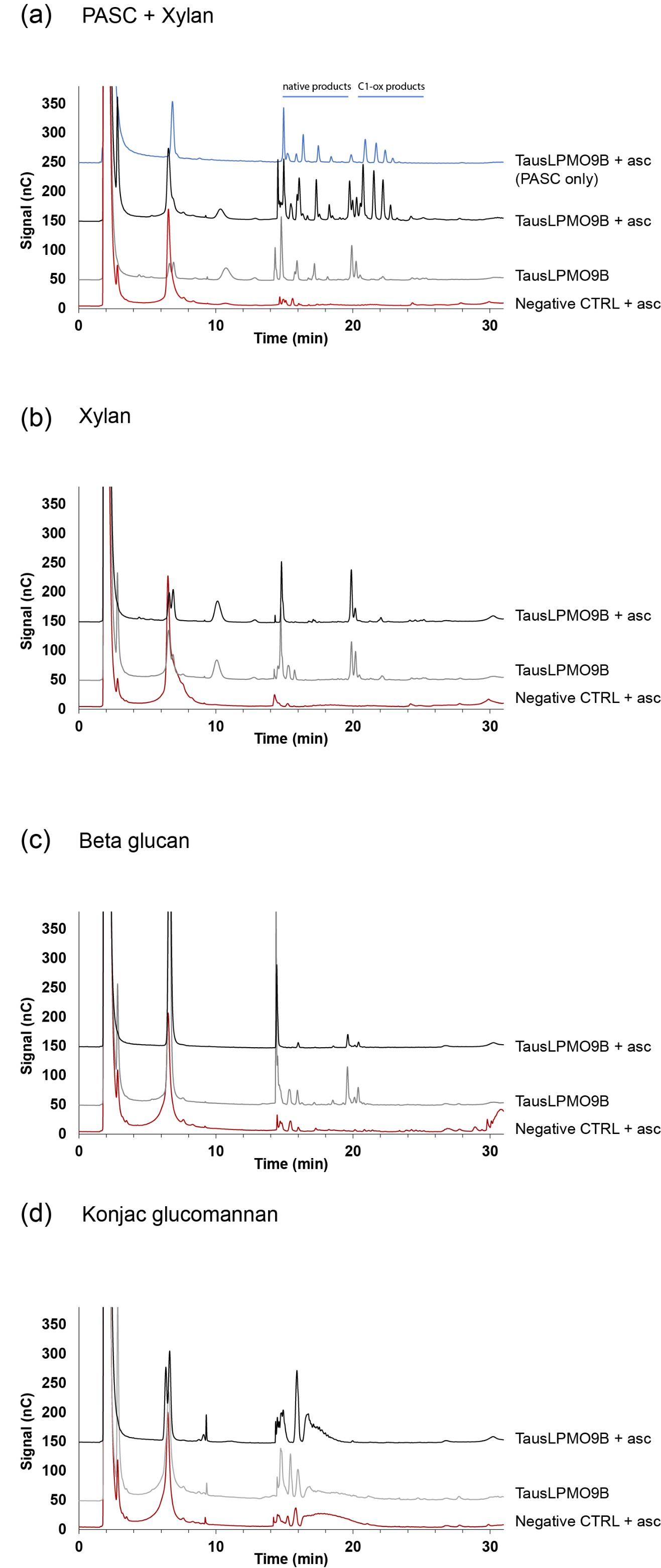
**

**Figure S7** Activity of *Taus*LPMO9B on different substrates. HPAEC-PAD chromatograms for products released after 24 h incubation of 1 µM enzyme at 45°C, pH 5.0, in the presence (black line) or absence (grey line) of 1 mM ascorbic acid. Negative control reactions did not contain any enzyme (red line). The reaction mixtures contained 0.1% (w/v) of different substrates: **a)** PASC (blue line) or a mixture of 0.1% (w/v) xylan and 0.1% (w/v) PASC, **b)** Xylan, **c)** Beta glucan, and **d)** Konjac glucomannan. No difference was detected upon incubation of enzyme with or without reductant. Additional experiments, not shown here, showed that *Taus*LPMO9B was not active towards cellopentaose and cellohexaose.

**b**

**a**

**Figure S8** Thermal stability of *Taus*LPMO9B analyzed with the Thermofluor assay. The graphs show melting curves of *Taus*LPMO9B at different pHs. The sample (10 µM protein) was heated with stepwise increments of 0.5°C per minute and a 30-s hold, and the fluorescence was monitored. Panel **a** shows fluorescence intensity as a function of the temperature; panel **b** shows the change in fluorescence intensity as a function of temperature. The derivative of the curve (in panel **b**) was used to obtain apparent melting temperatures.

# References

1. Borisova AS, Isaksen T, Dimarogona M, Kognole AA, Mathiesen G, Várnai A, et al. Structural and functional characterization of a lytic polysaccharide monooxygenase with broad substrate specificity. J Biol Chem. 2015;290(38):22955–69.

2. Harris P V, Welner D, Mcfarland KC, Re E, Poulsen JN, Brown K, et al. Stimulation of lignocellulosic biomass hydrolysis by proteins of glycoside hydrolase family 61 : structure and function of a large , enigmatic family. Biochemistry. 2010;49:3305–16.

3. Laurent CVFP, Sun P, Scheiblbrandner S, Csarman F, Cannazza P, Frommhagen M, et al. Influence of lytic polysaccharide monooxygenase active site segments on activity and affinity. Int J Mol Sci. 2019;20(24):6219.

4. Letunic I, Bork P. Interactive Tree Of Life v2: online annotation and display of phylogenetic trees made easy. Nucleic Acids Res. 2011;39:W475–8.
